# The translational network for metabolic disease – from protein interaction to disease co-occurrence

**DOI:** 10.1186/s12859-019-3106-9

**Published:** 2019-11-13

**Authors:** Yonghyun Nam, Dong-gi Lee, Sunjoo Bang, Ju Han Kim, Jae-Hoon Kim, Hyunjung Shin

**Affiliations:** 1 Department of Industrial Engineering, Ajou University, 206, World cup-ro, Yeongtong-gu, Suwon-si, Gyeonggi-do 16499 Republic of Korea; 2 Seoul National University Biomedical Informatics (SNUBI), Division of Biomedical Informatics, Seoul National University College of Medicine, 103, Daehak-ro, Jongno-gu, Seoul, 03080 Republic of Korea

**Keywords:** Semi-supervised learning, Disease network, Comorbidity, Protein interaction, Disease scoring

## Abstract

**Background:**

The recent advances in *human disease network* have provided insights into establishing the relationships between the genotypes and phenotypes of diseases. In spite of the great progress, it yet remains as *only a map of topologies between diseases,* but not being able to be a pragmatic diagnostic/prognostic tool in medicine. It can further evolve from a map to a *translational* tool if it equips with a function of scoring that measures the likelihoods of the association between diseases. Then, a physician, when practicing on a patient, can suggest several diseases that are highly likely to co-occur with a primary disease according to the scores. In this study, we propose a method of implementing ‘*n-of-1 utility’* (*n* potential diseases of *one* patient) to human disease network—*the translational disease network*.

**Results:**

We first construct a *disease network* by introducing the notion of *walk* in graph theory to *protein-protein interaction network*, and then provide a *scoring algorithm* quantifying the likelihoods of *disease co-occurrence* given a primary disease. Metabolic diseases, that are highly prevalent but have found only a few associations in previous studies, are chosen as entries of the network.

**Conclusions:**

The proposed method substantially increased *connectivity* between metabolic diseases and provided *scores* of *co-occurring diseases.* The increase in connectivity turned the disease network *info-richer*. The result lifted the AUC of random guessing up to 0.72 and appeared to be concordant with the existing literatures on *disease comorbidity*.

## Background

The recent advances in human disease networks have provided insights into establishing the relationships between the genotypes and phenotypes of human diseases [1–3]. A disease (or disorder) is often thought of as resulting from rare mutations that trigger disruptions in underlying cellular functions. However, it is far from sufficient to define diseases solely by a mutation in a single gene because they are influenced by the totality of the intricate molecular connections between numerous cellular components [4–7]. A series of successful experiments developed in network biology have been beneficial for the progress of human disease network analysis [8, 9], which includes various types of molecular connections such as networks of gene co-expression, transcriptional regulations, protein interactions, metabolic pathways, and so on [10]. In [11], the authors provide a good review on the main features and the pros-cons of the existing methods for the disease-related biomolecular networks. Also, a comprehensive compilation of known disease-disease association can be found from many web services such as the DiseaseConnect (http://disease-connect.org) [12].

An initiative challenge for human disease networks was suggested by Goh et al. [13], which attempts to identify disease associations based on the genes that diseases share. Most diseases were grouped into several clusters; in particular, the cancer cluster is tightly interconnected owing to the many genes associated with multiple types of cancer. This has led to successful research using various molecular networks. Zhang et al. (2011) proposed a disease network using the protein-protein interaction (PPI) network [14], motivated by studies indicating the genes that share similar or the same disease phenotypes tend to encode proteins that interact with each other [15, 16]. Indeed, the Hermansky–Pudlak syndrome [17] and Fanconi anemia [18] are known to be caused by mutations affecting different, but interacting, proteins. Lee et al. (2008) used the metabolic pathway network, and hypothesized that diseases are associated if they are linked to potentially correlated metabolic reactions [19]. Paik et al. (2014) proposed network-based disease–disease similarity analysis by focusing on topological similarity, suggesting disease-pathological symptoms through protein interactions [20]. A number of disease network studies have incorporated related methods [21–24].

Although our understanding of disease networks has expanded by virtue of the growth in theories and technical tools in the past, there is some room for improvement in the previous research. *First,* many works on disease networks have not identified tight associations for metabolic diseases. For cancer related diseases, a cancer cluster is successfully characterized and its associations to other diseases, including several diseases with a strong predisposition to cancer (such as Fanconi Anemia and Ataxia Telangiectasia), are well established because many genes are associated with multiple types of cancer (*TP53, KRAS, ERBB2, NF1,* etc.). However, for metabolic diseases, they are underrepresented, do not appear to form a distinct cluster, and have the fewest connections to other diseases. This result is attributed to the lack of information on genetic mutations associated with metabolic disease. But in practice, metabolic diseases may not really be so independent of one another, since certain metabolic diseases such as diabetes mellitus and hypoglycemia [25] or dyslipidemia and amyotrophic lateral sclerosis [26] often co-occur in the same individual and one can sometimes be considered a significant risk factor for the presence of the other. In the meantime, one can find another motivation for the research on metabolic disease network when consulting the report on prevalence statistics for diseases. A large proportion of people suffer from metabolic disruptions and the incidence rate is in trending upwards at the population level. During the period 2003 to 2012, metabolic syndrome prevalence in the United States was approximately, 18.0% among adults aged 20~39 years, 35.0% among adults aged 40~59 years, and 46.7% among adults aged 60 years and above [27]. With respect to diabetes in particular, 9.3% of the U.S. population has been diagnosed with diabetes, and 37% of U.S. adults aged 20 years or older were pre-diabetic in 2009–2012 [28]. On the other hand, the cancer incidence rate in 2011 was approximately 3.2% of the population and has declined year by year. The cancer death rate has also been declining by 1.5% per year for two decades. When comparing the statistics of metabolic diseases with those of cancer [29], one cannot lay less emphasis on the significance of metabolic diseases. Despite awareness of the importance, studies on the network analysis for metabolic diseases have not been yet well established. From those perspectives, it is of special interest to elucidate further the associations among metabolic diseases.

*Second*, disease networks have not been of benefit to medical research and practice yet, although they are poised to play a big role at the cellular level. The majority of the research on disease networks is still limited to developing a methodology to construct the network even when the approach makes use of sources of information from disease-gene associations, the interactions of proteins encoded by disease-related genes, or the metabolic pathways of diseases. We conjecture that it is because the research, in most cases, was issued and conducted by biologists pursuing purely scientific findings. In the perspective of physicians/clinicians/patients, however, this practice may be regarded as somewhat unkind since the results obtained from biology labs are too remote to be useful in the face of the reality of practicing medicine on patients. A doctor who is referencing a disease network when he/she treats a patient diagnosed with a particular disease may want to know if a co-occurrence is accidental or causal or if it increases the likelihood of the development of other diseases. It would be more convenient if the answer is given in the form of number, something like a score or a probability value, for disease co-occurrence. Unfortunately, most of the current disease networks do not provide quantified information such as disease co-occurrence scores, instead presenting a map of topologies between diseases. A recent study of Paik et al. (2014) provides the comorbidity information by comparing protein interaction-based disease network and medical reports-based disease network [20]. It gives a simple dichotomous information if two diseases are comorbid or not.

To address the limitations discussed above, we suggest a *disease network* model that provides quantified information, *scores* or *probabilities* for co-occurring diseases. To overcome sparse connectivity between metabolic diseases, we introduce the notion of *walk* of graph theory. The length of walk controls the range of protein-protein interactions that we use for identification of disease-disease associations. This idea is inspired by the definition of metabolic disease—the result of a genetic defect which frequently causes a metabolic enzyme to be non-expressed, inactive, or functionally compromised [30]. This implies that we may identify *latent* associations between metabolic diseases if we search deeper into the PPI network. There may be hidden evidences that seemingly unrelated proteins interact somewhere in any of metabolic pathways. We then propose a method for *disease scoring*. Scores are calculated based on graph-based semi-supervised learning (SSL). The algorithm collects the *latent* information spread over the disease network to calculate scores. We validate the results of the proposed method with comorbidity literatures providing enrichment study for disease co-occurrence.

## Results

### Data for constructing disease networks

To construct a network of metabolic diseases, a list was obtained from MeSH in 2017. The National Library of Medicine has a controlled vocabulary thesaurus in the form of a taxonomy. When considering up to the second level of the taxonomy, there are 302 descriptors for metabolic diseases out of the 4663 listed diseases.

On the other hand, disease-protein relationship and protein-protein interactions data were obtained from the eight existing interaction databases: 53,480 disease-protein relationships between 2411 diseases and 7733 proteins, and 60,794 protein-protein interactions among 15,281 proteins, respectively. Based on Entrez gene and MeSH, we have curated all relational information related to metabolic diseases from multiple databases. The presence or absence of disease-protein relation was established to produce a binary disease vector. Table 1 summarizes the data sources. Of 302 metabolic diseases, only 181 have at least one disease-protein relationship, therefore, the size (the number of nodes) of our disease network was set to 181. (Additional file 1: Table A1 in Appendix A provides a full list of the 181 diseases.) The size of the PPI network is 15,281. The configuration of the two networks will be described as the upper and lower layers in Fig. 5a.

**Table 1:** Data for diseases, disease-protein relationships, and protein-protein interactions, and literature for comorbidity analysis: the number in parentheses indicates the amount of data originating from the respective sources. See also Additional file 1: Appendix A and C

	Metabolic diseases	Disease-protein relationship	Protein-protein interaction	Comorbidity analysis
Data Sources	MeSH The Medical Subject Headings www.nlm.nih.gov/mesh/	CTD (7624) ver. 2014/07/11 Comparative Toxicogenomics Database, www.ctdbase.org/ GAD (34,773) ver.2013/07/16 Genetic Association Database, www.geneticassociationdb.nih.gov/ OMIM (4078) ver. 2014/03/10 Online Mendelian Inheritance in Man, www.omim.org/ PharmGKB (6610) ver. 2015/07/20 The Pharmacogenomics Knowledge Base, www.pharmgkb.org/ TTD (395) ver. 2013/07/04 Therapeutic Target Database, www.bidd.nus.edu.sg/group/cjttd/	DIP (773) ver. 2014/10/10 Database of Interacting Proteins, www.dip.doebi.ucla.edu/dip/Main.cgi Entrez Gene (58,778) ver. 2014/07/20 www.jura.wi.mit.edu/entrez_gene/ MINT (736) ver. 2014/07/08 Molecular Interaction Database, www.mint.bio.uniroma2.it/mint/ PharmGKB (507) ver. 2014/07/20 The Pharmacogenomics Knowledge Base, www.pharmgkb.org/	PubMed Literature US National Library of Medicine National Institutes of Health
Number of Data	181 out of 302 metabolic diseases	53,480 relations between 2411 diseases and 7733 proteins	60,794 interactions of 15,281 proteins	62 pairs of 55 diseases

### Results for disease networks from the PPI network

The 181 metabolic diseases are represented as 15,281 dimensional vectors, where a disease vector is composed of binary attributes, each of which stands for presence (‘1’) or absence (‘0’) of the association with a particular protein as in Fig. 6. A disease network is built by calculating associations between disease vectors. Cosine distance is employed and is fed into the Gaussian similarity function (1): cosine distance is defined as *dist* (***x***_*i*_, ***x***_*j*_) = 1 − ( ***x***_*i*_ · ***x***_*j*_)/(‖ ***x***_*i*_‖ · ‖ ***x***_*j*_‖), where *dist* (*x*_*i*_, *x*_*j*_) is transformed to similarity using cosine distance between *x*_*i*_ and *x*_*j*_, ‘∙’ indicates the vector dot product, and ‖***x***‖ is the length of vector ***x***.

**Fig. 1:**
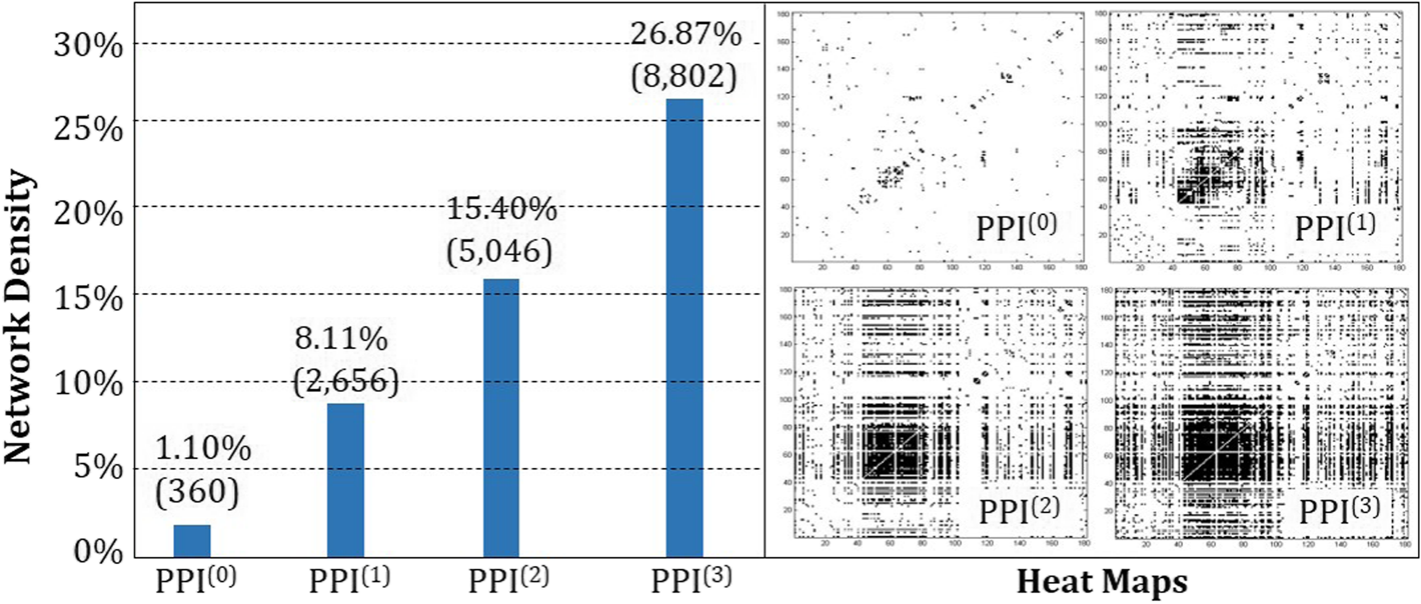
Changes in network density by a q-step walk on the PPI network: The amount of connections for disease-disease association increases as the step size q increases. With rich connections among diseases, the chances of having inferences for most diseases, including rare diseases, are increased because it enables us to use the information propagated from other diseases through the connections

A disease vector for metabolic diseases is sparse since only a few disease-protein associations are known. This implies that the resulting disease network has sparse connectivity as well: the density of the matrix for disease-disease association is only 1.10%. See Fig. 1. Note that this network corresponds to ***PPI***^(**0**)^ by the definitions in Methods section. In Fig. 1, the entry represents the counted number for disease-disease associations. The matrix connectivity increases as q increase. The heat-maps for disease-disease associations get denser as the step size ***q*** increases. The density reaches 26.87% in ***PPI***^(**3**)^. The results show that we can widen the range of association of a disease (when there is only little information available) by applying the notion of *q***-**step on the PPI network. And further, we can infer associations for most diseases and for rare diseases as well with the resulting disease network.

**Fig. 2:**
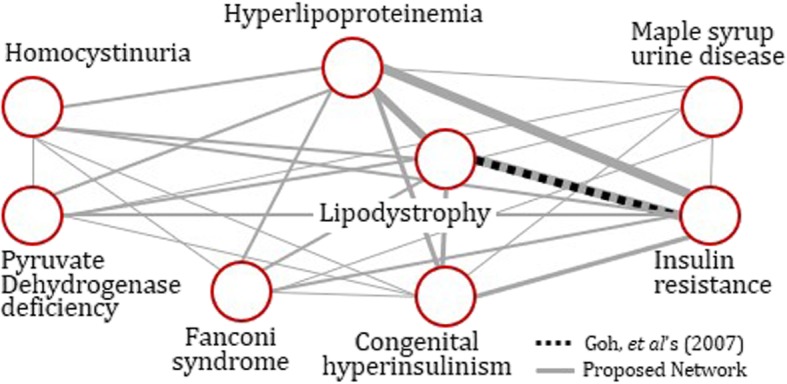
Disease network comparison: A small subset of diseases belonging to the metabolic diseases category in MeSH is selected. The nodes in the graph represent the eight selected diseases, and the width of an edge indicates connection strength proportional to the number of shared proteins between two diseases. In Goh et al.’s approach, there is only a single edge connecting two disease nodes, while the remaining six are disconnected. In contrast, in *PPI*^(0~*q*)^, all eight disease nodes are connected, and each has a high degree of node connectivity with various connection strengths

Figure 2 presents a toy demonstration for the proposed disease network vs. the existing one by Goh et al.’s. The disease network is composed of eight metabolic diseases such as hyperlipoproteinemia, homocystinuria, maple syrup urine disease, pyruvate dehydrogenase deficiency, lipodystrophy, insulin resistance, Fanconi syndrome, and congenital hyperinsulinism. The dotted line indicates the edge by Goh et al.’s: there is only a single edge connecting two diseases and the rest are disconnected. In contrast, in ***PPI***^(***integrated***)^ disease network (a network piling up ***PPI***^(**0**)^ up to ***PPI***^(**3**)^), the diseases are all connected, and the connection between the nodes is large with various connection strengths. The width of an edge is proportional to the number of shared proteins. These are shown in grey solid lines in Fig. 2. The comparison results show that we are now able to make inferences about most diseases (including rare diseases) based on these richer connections of the disease network. Additional file 1: Fig. B1 in Appendix B provides a full network comparison. (The Matlab source code is available in Additional file 2: ‘NetworkConstruction.m’.)

### Results for disease scoring

#### Enrichment with comorbidity study

To validate the proposed method as reliable in real medical/clinical practice, we adopted data from the literature of a comorbidity study as an independent source of information for this test. Comorbidity measures the presence of one or more additional diseases (or disorders) co-occurring with a primary disease or the effects of such additional diseases. The sources of information, used by the researchers and scientists working on the question of comorbidity, are case histories [31, 32], hospital records of patients [33] and other medical documentation kept by family doctors, or insurance companies [34]. Therefore, data in the literature from comorbidity studies are mainly based on the clinical experience and qualifications of the physicians carrying out clinical, instrumental, and laboratory-confirmed diagnoses. In total, 62 pairs of 55 comorbid diseases were obtained from a literature survey. See the last column of Table 1. The full list of the comorbidity literature used in this study is provided in Table C1, and the references are provided in Additional file 1: Appendix C.

#### Comparative experiments for scoring

The proposed SSL based scoring method (in Methods section) was applied to ***PPI***^(***q***)^ ‘s, hypothesizing that our extended disease networks will provide improved scoring as compared to the existing disease network produced by Goh et al. Note that Goh et al.’s approach coincides with that of our ***PPI***^(**0**)^ network. In order to obtain a random control as a reference point, we created a randomized network; we shuffled disease-disease associations while keeping both the number of connected nodes and the degree of a node the same as those of ***PPI***^(***integrated***)^, and projected it onto a disease network. The loss-smoothness tradeoff parameter *μ* in (5) was set to a large number (*μ* = 100). From the published comorbidity studies, 55 out of 181 diseases were able to obtain labels in our SSL scoring model. For instance, by setting the label of a disease on ‘ *y*_*l*_ = 1 ’, while keeping unchanged the labels of the remaining 180 diseases as ‘ *y*_*u*_ = 0 ’, we obtained the predicted score for identifying the comorbid diseases with the given disease. The experiment was repeated 55 times and the performance was measured by AUC (the area under the receiver operating characteristic curve) [35]. The AUC was obtained by comparing predictive value ***f*** in (5) and PubMed Literatures: presence (‘1’) or absence (‘0’) of PubMed literatures. It is used as a standard for disease association and comorbidity disease.

**Fig. 3:**
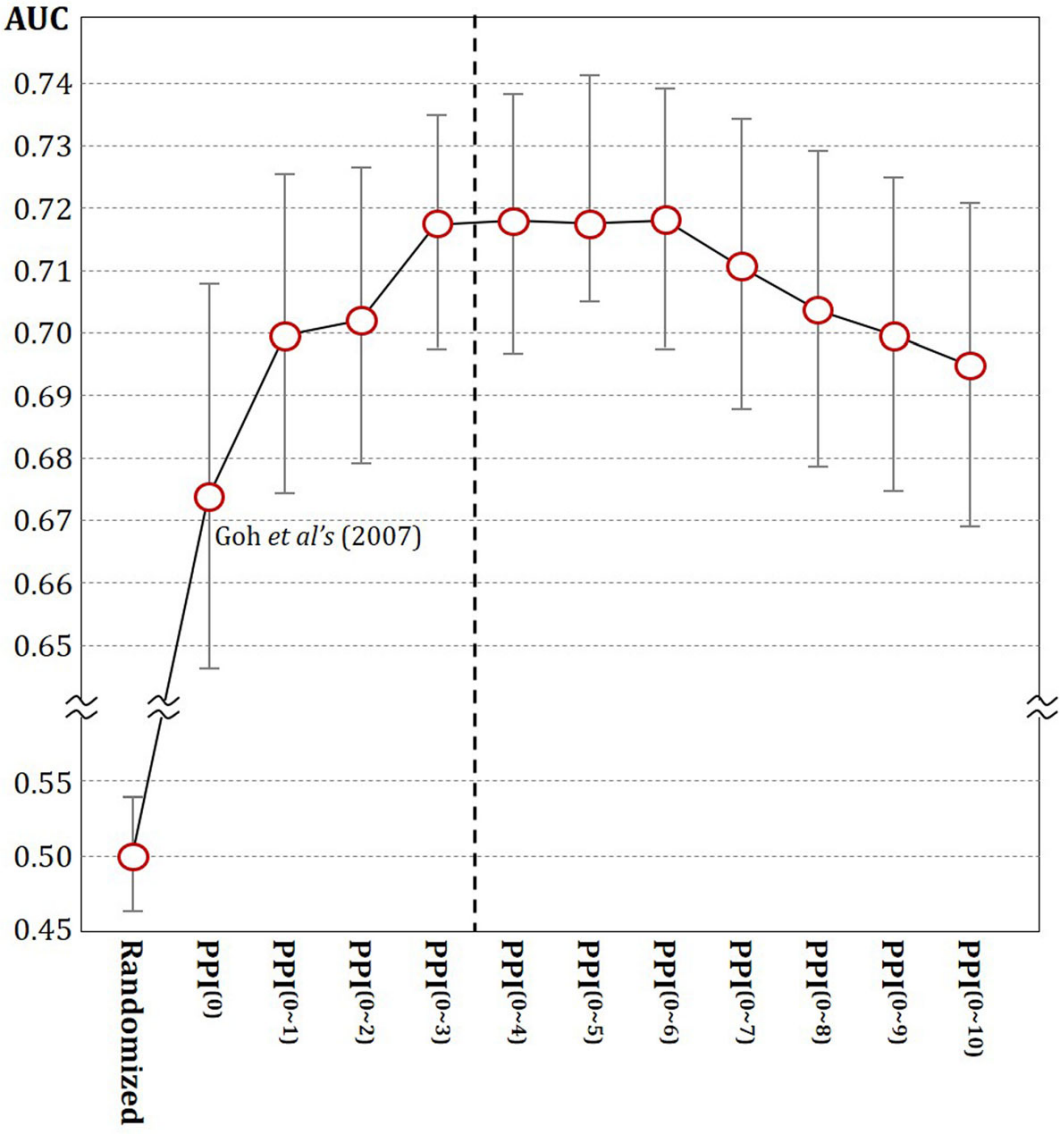
AUC comparison from *q* = 0 to 10 for integrated networks: *PPI*^(0~*q*)^. The experiment was repeated 55 times, and the average AUC value with the standard deviation is presented as a circle with an error bar. *PPI*^(0)^, *PPI*^(1)^, *PPI*^(2)^, and *PPI*^(3)^ are individual disease networks constructed as described in Methods section. *PPI*^(0~q)^ s are integrated networks from Eq. (6). *PPI*^(0)^ corresponds to the existing disease network suggested by Goh et al. [21]. A randomized network is added to our experiment to obtain a reference performance. The best performance was achieved by *PPI*^(0~3)^, and *p*-values of the pairwise t-tests are shown in the bottom of the plot

The AUC performances for the disease networks are summarized in Fig. 3. The randomized disease network produces an AUC of 0.5, which is only equivalent to the performance by random guessing. However, ***PPI***^(**0**)^ lifts the performance up to 0.69. Thereafter, the performance of the disease network continuously increases as the step size *q* increases up to *q* = 3: ***PPI***^(**0**)^ < ***PPI***^(**1**)^ < ***PPI***^(**2**)^ < ***PPI***^(**3**)^. Given these results, it is plausible that the more connected a disease network, the higher the AUC performance will be. After *q* = 3, however, the performance stayed unchanged till *q* = 6, then begins to degrade. We conjectured that the complexity of ***PPI***^(**3**)^, in terms of network connectivity, is enough to draw most of information in data, therefore more complication may not be needed. Additional file 1: Fig. D1 in Appendix D shows performance of the individual networks. On the other hand, it is interesting to observe that combining ***PPI***^(***q***)^ to ***PPI***^(***q +*** **1**)^ (*q* = 0, 1, 2) further increases the AUC performance. ***PPI***^(**0*~*1**)^ is an integrated network piling up ***PPI***^(**0**)^ onto ***PPI***^(**1**)^, and ***PPI***^(**0*~*2**)^ is similarly constructed. This pattern of improvement ends with ***PPI***^(**0*~*3**)^, resulting in an AUC of 0.72. The further integration did not make significant improvement in performance, so it was decided as our best network. The *p*-values of the pairwise *t*-test (Additional file 1: Table D1 in Appendix D) demonstrate the statistical significance of the improved performance of ***PPI***^(**0*~*3**)^ over the others. Additional experiments were conducted to verify the performance instead of leave-one-out. 5-fold cross validation was performed for three disease groups: metabolic diseases, neoplasms, and nervous system diseases. The experiment was repeated ten times, and for each of them, 5CV was conducted after random permutation of data. The results were summarized in an Additional file 1: Appendix E.

## Discussion

### Implication of the probabilities of the associated diseases

**Fig. 4:**
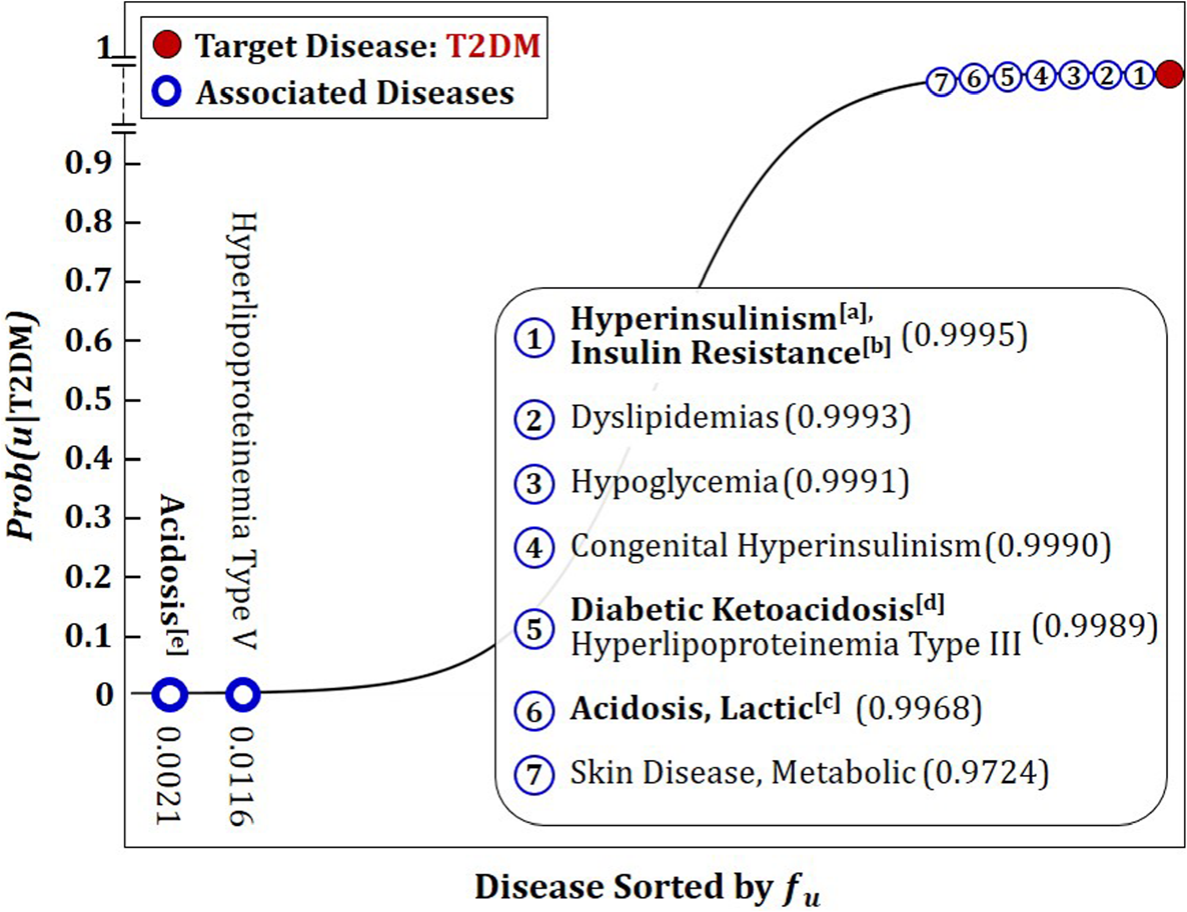
Probabilities of the diseases associated with T2DM: The solid line represents the probability values of the 180 diseases. Shown on the line with open circles are the locations of 13 diseases comorbid with diabetes mellitus type II. The probability values support the knowledge based on real medical practice and vice versa

Figure 4 depicts a typical example of the proposed scoring results with the probabilistic transfer function (6). Diabetes mellitus type II (T2DM) was set as the labeled (target) disease, and then the probability values for association with the remaining 180 diseases were obtained from the integrated network, ***PPI***^(**0*~*3**)^. The solid line in the figure stands for the probability values of the 180 diseases. The open circles on the line correspond to the diseases comorbid with T2DM evidenced by the literature. The below shows the clinical implications for some of the marked comorbid diseases observed in the literature. More evidences can be found in Additional file 1: Appendix F.

#### High in insulin resistance^[a]^ and hyperinsulinism^[b]^

T2DM is preceded or accompanied by an elevated adiposity that causes insulin resistance [36]. Insulin resistance causes hyperinsulinemia to maintain normal glucose levels. When the pancreas cannot sustain hyperinsulinemia to overcome insulin resistance, pre-diabetes or T2DM ensues [37].

#### High in lactic acidosis^[c]^

Lactic acidosis is caused by accumulation of lactic acid more rapidly than it can be metabolized. It may occur spontaneously or in association with diseases such as T2DM, leukemia, or liver failure [38].

#### High in diabetic ketoacidosis^[d]^ vs. low in acidosis^[e]^

Presence of Diabetic ketoacidosis has been increasingly recognized in patients with T2DM, and a newer entity called ketosis-prone diabetes is also commonly recognized [39, 40]. Diabetic ketoacidosis in patients with T2DM tends to present with a less severe acidosis and patients are more likely to have normal potassium levels [41–43].

## Conclusion

In this study, we proposed two novel methods: the method of disease network construction by extracting latent information from different depths of layers of the PPI network and the method of disease scoring based on SSL by collecting the latent information spread over the network. To examine whether the proposed method for disease co-occurrence provides predictions within reasonable bounds in practice, we investigated pairs of comorbid diseases that were reported in the literature and compared them with the obtained scores. The result was promising; the scoring results appear concordant with conventional comorbidity studies.

There are some noteworthy features of the present study: (a) Despite great progress in research on disease networks, there are still barriers for physicians to use it in practice: a disease network has been little more than a map of topologies between diseases. It is inconvenient to deduce the co-occurrence of the associated diseases, and they do not have enough confidence to put it into action when practicing a patient. From this point of view, this study suggests a streamlined methodology with biologically driven knowledge—the protein-protein interaction data— and the scores for disease co-occurrences. This will eventually assist physicians in adopting smarter strategies earlier to aid them in tackling the numerous intricacies inherent to the treatment of diseases.

(b) The proposed method of constructing a disease network is an unprecedented and systematic approach. Previous work identifies disease-disease associations based on the gene that diseases share [13], based on the gene encoding the protein that interacts with the protein encoded by other disease genes [14], or based on shared metabolites and correlated metabolic reactions [19]. The present study provides a framework embracing the previous work: Goh et al. (2007)‘s is analogous to our ***PPI***^(**0**)^, Zhang et al. (2011)‘s to ***PPI***^(**0*~*1**)^, Lee et al. (2008)‘s to ***PPI***^(***q***)^ where *q* is arbitrary depending on the size of metabolic pathway, and Paik et al. (2014)‘s to ***PPI***^(***q***)^ where *q* is 0 and 1. With our methodology, it becomes simple to expand or shrink the reference ranges of the PPI network—just adjust the step size *q* of walk! With the ongoing growth in study for the completeness of the PPI network such as [44] and the references therein, we expect that the proposed methodology on how to construct a disease network from PPI will further power the disease mechanism research.

(c) An algorithm for disease scoring must be able to deal with the circumstance that only limited labeled data are provided because a patient will provide only a few pieces of information on one or two diseases that he/she has contracted. SSL can handle these situations, and this trait has inspired us to develop a scoring algorithm based on SSL.

In this study, we have only focused on the connectivity of diseases via genes without considering they are essential or redundant. We could not disregard a gene even if it is regarded as redundant because it can be used as a link to reach other diseases. We provided a typical case about how a disease is linked to other diseases, and therein, which gene turns out to be important (essential) for the connection (See Additional file 1: Appendix G). We note that the current study is motivated by and developed for metabolic diseases that are known to be the most disconnected class for most human disease networks. Extension of this methodology to the whole set of human diseases should be attempted next. We added some preliminary results for other categories of disease to Additional file 1: Appendix H. Another extension may be developed by incorporating diverse data sources to our method. Particularly, if updating the translational disease network with cleaner and higher quality of data [45], we expect that it will be upgraded to be more reliable and accurate.

## Methods

**Fig. 5:**
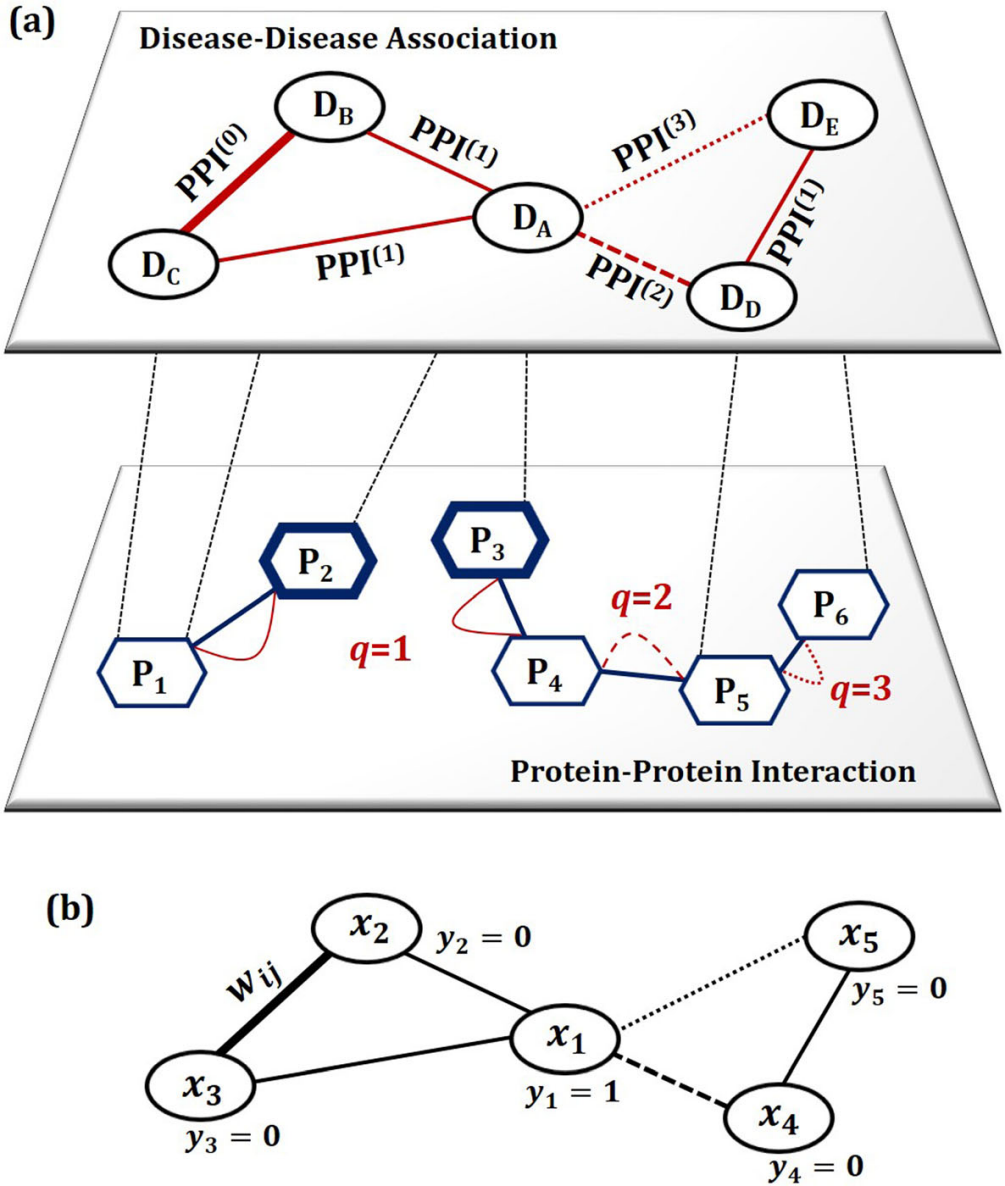
The proposed model: **a** a method of constructing a disease network based on a *q*-step walk on the protein-protein interaction (PPI) network and **b** a scoring model for calculating the scores of disease co-occurrence when a specific disease is given

The translational disease networks consist of two methods: (a) *a method of constructing a disease network* based on protein-protein interaction data and (b) *a network-based scoring method* for calculating the probabilities of disease co-occurrence when a specific disease is given. The resulting disease network from the first becomes the base on which the second works. See (a) and (b) in Fig. 5.

### Disease networks by q-step walks on the PPI network

**Fig. 6:**
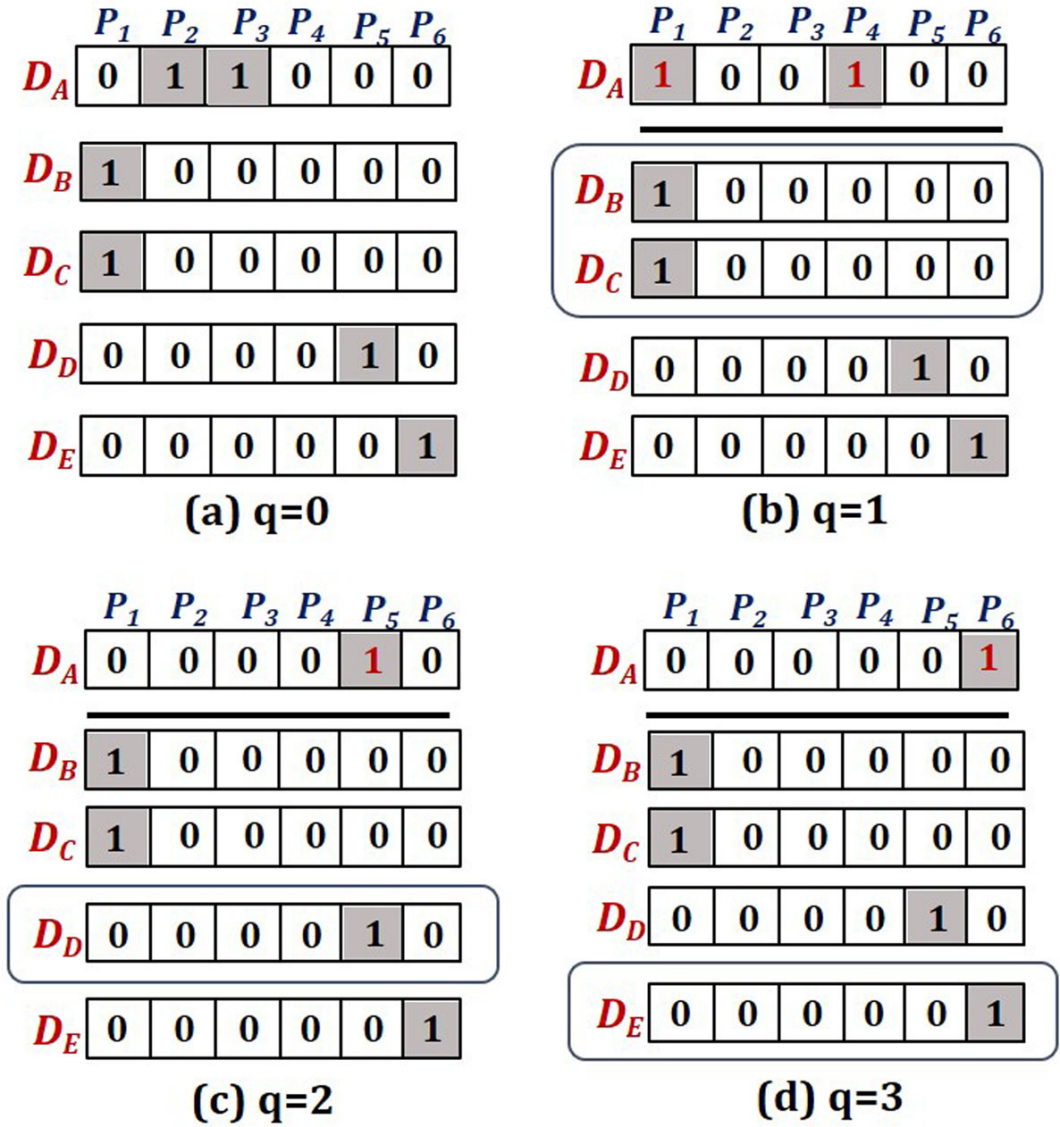
Associated diseases of *D*_A_ by *q*-step walks on the PPI network in the case of **a** *q*=0, **b** *q*=1, **c** *q*=2 and **d** *q*=3

The disease network is a graph, *G*(*V*, *W*), that represents the associations between pairs of diseases by assigning a weight to the edge connecting two diseases. In the network, the node *V* denotes diseases and the weight *W* denotes similarity between the sequences of proteins that two diseases commonly share. In the proposed method, the notion of *commonly shared proteins* is expanded by applying the *walk* (or *path*) of graph theory to the PPI network. The step size of walk on the PPI network determines the number of shared proteins. On a graph, a ‘*walk*’ starting at node *v*_*A*_ and ending at node *v*_*B*_, is represented as (*v*_*A*_ → *v*_1_ → … → *v*_*n*_ → *v*_*B*_). The edges connect the successive nodes in a walk. Let us define a ‘*q*-*step walk*’ as a walk of length *q*, which travels *q* edges departing from *v*_*A*_ for *v*_*B*_. In a conventional approach for constructing a disease network, the two diseases are defined as *associated* only if they are known to share same proteins. In Fig. 5a, *D*_B_ and *D*_C_ share protein *P*_1_, therefore they are considered as associated. Figure 6a rephrases this in disease-protein vector representation, and we see that there exists no other disease association than a single one between *D*_B_ and *D*_C_. In terms of *q*-step walk, it corresponds to the case of *q* = 0. However, if the notion of *q*-step walk is applied to the PPI network, disease associations can be further expanded. Consider *D*_A_ in Fig. 5a which is known to be related to two proteins *P*_2_ and *P*_3_. And it is not associated with any of diseases in 0-step walk. An 1-step walk departing from those proteins reaches *P*_1_ and *P*_4_, respectively, (see Fig. 5a), and produces a different disease-protein vector (see Fig. 6b). Then, it now can be associated with *D*_B_ and *D*_C_. Similarly, a 2-step walk ( *P*_3_ → *P*_4_ → *P*_5_) or a 3-step walk (*P*_3_ → *P*_4_ → *P*_5_ → *P*_6_) makes expanded associations with other diseases, which are described in Fig. 6c and d.

Applying different *q*-step walk to the PPI network, a disease network can be differently constructed, and is denoted as ***PPI***^(*q*)^. The following lists general definitions of ***PPI***^(*q*)^ ‘s where *q* = 0, 1, 2, 3**.** The disease network by *q*-step walks on PPI network is a graph *PPI*^(*q*)^ = *G*(*V*, *W*^(*q*)^), where *W*^(*q*)^ is the similarity after applying *q*-step walks. (Note that the initial network, ***PPI***^(0)^, is original disease network *G*(*V*, *W*)). Consider two diseases, Disease I and Disease II, assuming that the former is known to be associated with protein *P*_I_ and the latter with *P*_II_. The step-by-step process of *q*-step walk is described in detail in Additional file 1: Appendix I.

- ▪ ***PPI***^(0)^: The two diseases are defined as *associated* only if *P*_I_ and *P*_II_ are identical (*P*_I_ ≡ *P*_II_)—the disease network by *0-step walk*.
- ▪ ***PPI***^(1)^: An association between the two diseases is defined when the proteins are known to *interact* (*P*_I_~*P*_II_ where ‘~’ stands for interaction)— the disease network by *1-step walk*.
- ▪ ***PPI***^(2)^: Let us introduce an extra protein *P*_III_ which interacts with the two disease proteins (*P*_III_~*P*_I_, *P*_III_~*P*_II_). Through bypassing the medium protein, *P*_I_ → *P*_III_ → *P*_II_ and vice versa, the two diseases are related— the disease network by *2-step walk.*
- ▪ ***PPI***^(3)^: Similarly, a *3-step walk* on the PPI network, *P*_I_ → *P*_III_ → *P*_IV_ → *P*_II_, can bridge the two diseases via two medium proteins, *P*_III_ and *P*_IV_—the disease network by *3-step walk.*

One may further develop ***PPI***^(*q*)^ ‘s by increasing the walk length *q*. As *q* increases, more associations can be found among diseases, and the density of the disease network increases. It is conjectured that a network of a larger step size is relatively less informative because of loss of information while touring around the network. On the contrary, a small step size *q* provides a good quality of associations between diseases but it may result in a disconnected disease network where many of the diseases remain isolated (or dangled), and is described in this paper as a drawback of the status quo approaches [13, 14]. Note that disease associations based on the above definitions are reciprocal; hence, a resulting disease network, ***PPI***^(*q*)^, is symmetric.

### A scoring model for co-occurring diseases

Once a disease network is obtained, a person who has caught/contracted a particular disease may wish to be informed regarding *how likely he/she is to be exposed to other diseases*. Hypothesizing that *at least* one disease is known, the proposed *scoring* method calculates probabilities for the associated diseases.

#### Scoring algorithm

Let us define *disease scoring* as a function that quantifies the degree of commitment of the associated diseases when one or a few diseases are given. To embody scoring in a disease network, ***PPI***^(*q*)^, we employ the conventional settings for the graph-based semi-supervised learning (graph-based SSL) classification and modify it to be suitable for our scoring problem. SSL has attracted the interests of many researchers in areas where labeled data are a few but unlabeled data are abundant [5, 46–52]. And it has been reported that SSL successfully improves classification performance by supporting classifiers with unlabeled data [5]. The motivation of SSL is appealing for our problem because it will be typical for a patient to have contracted one or a few diseases (labeled data) but not the rest of the diseases (unlabeled data). To implement an SSL based scoring algorithm one has to perceive the difference between classification and scoring. In a (binary) classification problem, the labels given to a classifier are binary (+ 1 or − 1), and the resulting prediction is made by the way that each of the unlabeled data are assigned to either one class (+ 1) or the other (− 1). On the contrary, in a scoring problem, unary labels (+ 1) are given to a scorer, and the resulting predictions are scores that prioritize the unlabeled data for the given labels. In the proposed method, it also outputs the corresponding probability values. Figure 5b schematically describes our graph-based SSL scoring method, and the following paragraphs explain the details.

Consider a graph *G* = (*V*, *W*) with node *V* corresponding to the *n*(=*n*_*l*_ + *n*_*u*_) data points from labeled set ${S}_L=\left\{{\left({x}_i,{y}_i\right)}_{i=1}^{n_l}\right\}$ and unlabeled set ${S}_U=\left\{{\left({x}_i\right)}_{i={n}_l+1}^n\right\}$. In the proposed SSL based scoring problem, the *n*_*l*_ nodes are set to a unary label ***y***_*l*_ ∈ {1} while the unlabeled *n*_*u*_ nodes are set to zero (***y***_*u*_ ∈ {0}). The task is to assign scores ${\boldsymbol{f}}_u^T={\left({f}_{n_l+1},\dots, {f}_n\right)}^T$ on nodes *V*_*U*_. To compute a real-valued scoring function *f* : *V* → *ℝ* on *G*, one strategy is to let the label information propagate to the unlabeled nodes through edges *W*. The edge weight *w*_*ij*_ between the two nodes *x*_*i*_ and *x*_*j*_ can take a value of 0 or 1 in the simplest case. Usually, *dist* (*x*_*i*_, *x*_*j*_) between *x*_*i*_ and *x*_*j*_ is transformed to similarity using Gaussian

1 $$ {w}_{ij}=\left\{\begin{array}{cc}{\exp}^{- dist\ \left({x}_i,{x}_j\right)/{\sigma}^2}\ & if\ i\sim j\\ {}0& otherwise\end{array}\right. $$

where *i*~*j* indicates that the two nodes are connected. The connection strength is encoded in *w*_*ij*_ of a similarity matrix *W*, and a large value of *w*_*ij*_ represents more similarity between the two nodes. Assuming that *f*_*i*_ should be close to the given label *y*_*i*_ in labeled nodes—loss condition, and overall, *f*_*i*_ should not be too different from the *f*_*j*_ of adjacent nodes—smoothness condition, one can obtain ***f*** by minimizing the following quadratic functions:

2 $$ \underset{\boldsymbol{f}}{\min }\ H\left(\boldsymbol{f}\right)={\left(\boldsymbol{f}-\boldsymbol{y}\right)}^T\left(\boldsymbol{f}-\boldsymbol{y}\right)+{\mu \boldsymbol{f}}^T\boldsymbol{Lf} $$

where $\boldsymbol{y}={\left[{y}_1,\dots, {y}_{n_l},0,\dots, 0\right]}^T.$ The matrix ***L*** known as the graph Laplacian matrix, is defined as ***L*** = ***D*** − ***W*** where ***D*** =  *diag* (*d*_*i*_), *d*_*i*_ = $\sum \limits_j{w}_{ij}$. The user parameter *μ* trades off loss (the first term of *H*(.)) and smoothness (the second term). The solution of this problem becomes

3 $$ \boldsymbol{f}={\left(\boldsymbol{I}+\mu \boldsymbol{L}\right)}^{-1}\boldsymbol{y} $$

One may compute the scores for the unlabeled nodes explicitly in a block-wise representation of the similarity matrix *W* = [*W*_*ll*_ *W*_*lu*_ | *W*_*ul*_ *W*_*uu*_]. Let us represent (3) as a block structure and rearrange ***y*** to the left side of the equality,

4 $$ \left[\begin{array}{c}{\boldsymbol{y}}_l\\ {}{\boldsymbol{y}}_u\end{array}\right]=\left[\begin{array}{cc}\boldsymbol{I}+\mu \left({D}_{ll}-{W}_{ll}\right)& -\mu {W}_{lu}\\ {}-\mu {W}_{ul}& \boldsymbol{I}+\mu \left({D}_{uu}-{W}_{uu}\right)\end{array}\right]\left[\begin{array}{c}{\boldsymbol{f}}_l\\ {}{\boldsymbol{f}}_u\end{array}\right]. $$

Then, one can simplify (4) by substituting ***f***_*l*_ = ***y***_*l*_ and ***y***_*u*_ = **0**, and by writing it in terms of ***f***_*u*_, we obtain the scores for the unlabeled nodes,

5 $$ {\boldsymbol{f}}_u=\mu {\left\{\boldsymbol{I}+\mu \left({D}_{uu}-{W}_{uu}\right)\right\}}^{-1}{W}_{ul}{\boldsymbol{y}}_l. $$

On a network of diseases, by setting ***y***_*l*_ = 1 to the nodes for the contracted diseases, one can obtain scores ***f***_*u*_ for the rest of diseases from (5).

#### Probability calculation

After obtaining scores from a disease network, the next step is to transform the scores to probability values of disease co-occurrence. The resulting scores ***f***_*u*_ from (5) is unique and satisfies 0 < ***f***_*u*_ < 1 . After normalizing the overall ***f***_*u*_ ‘s, the scores can be associated with probability values as below

6 $$ Prob\left(u|l\right)=\frac{1}{1+{\mathit{\exp}}^{-{\boldsymbol{f}}_u/{\sigma}_f}} $$

where *σ*_*f*_ is a scale parameter. Given primary diseases (*l*), the output value of (6) measures *probability values* of *disease co-occurrence* for other diseases (*u*). One can refer to the values when attempting to figure out the secondary or tertiary diseases to the given one.

## Supplementary information

**Additional file 1.** Supplementary materials for the proposed method and results.

**Additional file 2.** The matlab source code for disease network construction from PPI network.

## Data Availability

The data can be found in PharmDB (http://pharmdb.org/). PharmDB is a tripartite pharmacological network database of human diseases, drugs, and proteins which compiles and integrates nine existing interaction databases (Access date: 2016. 11. 03).

## Abbreviations

CTD: Comparative Toxicogenomics Database
DIP: Database of Interacting Proteins
GAD: Genetic Association Database
MeSH: The Medical Subject Headings
MINT: Molecular Interaction Database
OMIM: Online Mendelian Inheritance in Man
PharmGKB: The Pharmacogenomics Knowledge Base
TTD: Therapeutic Target Database
