# Supplementary material for: The translational network for metabolic disease – from protein interaction to disease co-occurrence
Source: BMC Bioinformatics. 2019 Nov 13;20:576. doi: 10.1186/s12859-019-3106-9 (PMC6854734; doi:10.1186/s12859-019-3106-9)
Supplement: Supplementary file 2 — Additional file 2. The matlab source code for disease network construction from PPI network. [file 12859_2019_3106_MOESM2_ESM.pdf]

[APPENDICES]

The Translational Network for Metabolic Disease—from Protein Interaction to Disease Co-occurrence

Yonghyun Nam[1,†], Dong-gi Lee[1], Sunjoo Bang[1], Ju Han Kim[2], Jae-Hoon Kim[1,*], Hyunjung Shin[1,†,*]

[1]Department of Industrial Engineering, Ajou University, 206 Worldcup-ro, Yeongtong-gu, Suwon 16499, South Korea
[2]Seoul National University Biomedical Informatics (SNUBI), Div. of Biomedical Informatics, Seoul National University College of Medicine, Seoul 110799, South Korea

†Equally Contributed

*To whom correspondence should be addressed.

< Supplements >

- **Appendix A: 181 Metabolic Diseases**
- **Appendix B: Comparison with an Existing Disease Network**
- **Appendix C: Comorbidity Diseases**
- **Appendix D: Performance comparison with single and integrated network**
- **Appendix E: Comparative results for scoring**
- **Appendix F: More Examples on Associated Diseases by Scoring Results**
- **Appendix G: Connected Genes and Diseases of Gout**
- **Appendix H: Preliminary Results of Scoring for Other Target Diseases**
- **Appendix I: Constructing Disease Network by q-step walk**

Contact: shin@ajou.ac.kr

[Appendix A] Table A1. 181 Metabolic Diseases

1	Amyloidosis, Familial	62	Prediabetic State	123	Aspartylglucosaminuria
2	Achlorhydria	63	Glycosuria, Renal	124	Cystinosis
3	Acidosis	64	Hyperglycemia	125	Pycnodysostosis
4	Acidosis, Lactic	65	Glucose Intolerance	126	Metal Metabolism, Inborn Errors
5	Acidosis, Renal Tubular	66	Hyperinsulinism	127	Hypophosphatasia
6	Alkalosis	67	Congenital Hyperinsulinism	128	Hypophosphatemia, Familial
7	Brain Diseases, Metabolic	68	Insulin Resistance	129	Paralyses, Familial Periodic
8	Brain Diseases, Metabolic, Inborn	69	Hypoglycemia	130	Acatalasia
9	Cerebral Amyloid Angiopathy, Familial	70	Iron Metabolism Disorders	131	Adrenoleukodystrophy
10	Galactosemias	71	Anemia, Iron-Deficiency	132	Chondrodysplasia Punctata, Rhizomelic
11	Hartnup Disease	72	Iron Overload	133	Mevalonate Kinase Deficiency
12	Hepatolenticular Degeneration	73	Hemochromatosis	134	Refsum Disease
13	MELAS Syndrome	74	Lipid Metabolism Disorders	135	Refsum Disease, Infantile
14	Homocystinuria	75	Dyslipidemias	136	Zellweger Syndrome
15	Hyperglycinemia, Nonketotic	76	Hyperlipidemias	137	Porphyrias
16	Hyperlysinemias	77	Hypolipoproteinemias	138	Porphyria, Erythropoietic
17	Leigh Disease	78	Smith-Lemli-Opitz Syndrome	139	Porphyrias, Hepatic
18	Lesch-Nyhan Syndrome	79	Lipid Metabolism, Inborn Errors	140	Progeria
19	Pyruvate Carboxylase Deficiency Disease	80	HIV-Associated Lipodystrophy Syndrome	141	Purine-Pyrimidine Metabolism, Inborn Errors
20	Hereditary Central Nervous System Demyelinating Diseases	81	Pyruvate Dehydrogenase Complex Deficiency Disease	142	Dihydropyrimidine Dehydrogenase Deficiency
21	Menkes Kinky Hair Syndrome	82	Lipodystrophy, Familial Partial	143	Gout
22	MERRF Syndrome	83	Lipidoses	144	Renal Tubular Transport, Inborn Errors
23	Oculocerebrorenal Syndrome	84	Neuronal Ceroid-Lipofuscinoses	145	Fanconi Syndrome
24	Peroxisomal Disorders	85	Sjogren-Larsson Syndrome	146	Gitelman Syndrome
25	Phenylketonurias	86	Sphingolipidoses	147	Liddle Syndrome
26	Lipodystrophy	87	Lipomatosis	148	Pseudohypoaldosteronism
27	Maple Syrup Urine Disease	88	Xanthomatosis	149	Renal Aminoacidurias
28	Tyrosinemias	89	Xanthomatosis, Cerebrotendinous	150	Steroid Metabolism, Inborn Errors
29	Urea Cycle Disorders, Inborn	90	Malabsorption Syndromes	151	Adrenal Hyperplasia, Congenital
30	Hepatic Encephalopathy	91	Celiac Disease	152	Antley-Bixler Syndrome Phenotype
31	Kernicterus	92	Hyperhomocysteinemia	153	Ichthyosis, X-Linked
32	Mitochondrial Encephalomyopathies	93	Lactose Intolerance	154	Mitochondrial Diseases
33	Carbohydrate Metabolism, Inborn Errors	94	Ophthalmoplegia, Chronic Progressive External	155	Carbamoyl-Phosphate Synthase I Deficiency Disease
34	Calcinosis	95	Metabolic Syndrome X	156	Cytochrome-c Oxidase Deficiency
35	Nephrocalcinosis	96	Metabolism, Inborn Errors	157	Friedreich Ataxia
36	Hypercalcemia	97	Amino Acid Metabolism, Inborn Errors	158	Mitochondrial Myopathies
37	Hypocalcemia	98	Albinism	159	Steatorrhea
38	Pseudohypoparathyroidism	99	Alkaptonuria	160	Optic Atrophy, Autosomal Dominant
39	Pseudopseudohypoparathyroidism	100	Propionic Acidemia	161	Optic Atrophy, Hereditary, Leber
40	Rickets	101	Metabolic Diseases	162	Hypophosphatemia
41	Osteomalacia	102	Amyloid Neuropathies, Familial	163	Coproporphyria, Hereditary
42	DNA Repair-Deficiency Disorders	103	Calcium Metabolism Disorders	164	Porphyria, Acute Intermittent
43	Ataxia Telangiectasia	104	Congenital Disorders of Glycosylation	165	Porphyria Cutanea Tarda
44	Bloom Syndrome	105	Fructose Metabolism, Inborn Errors	166	Porphyria, Hepatoerythropoietic
45	Cockayne Syndrome	106	Fucosidosis	167	Porphyria, Variegate
46	Colorectal Neoplasms, Hereditary Nonpolyposis	107	Glucosephosphate Dehydrogenase Deficiency	168	Protoporphyria, Erythropoietic
47	Fanconi Anemia	108	Glycogen Storage Disease	169	Amyloidosis
48	Li-Fraumeni Syndrome	109	Hyperoxaluria, Primary	170	Amyloid Neuropathies
49	Nijmegen Breakage Syndrome	110	Mucolipidoses	171	Cerebral Amyloid Angiopathy
50	Rothmund-Thomson Syndrome	111	Pyruvate Metabolism, Inborn Errors	172	Amyotrophic Lateral Sclerosis
51	Severe Combined Immunodeficiency	112	Hyperbilirubinemia, Hereditary	173	Frontotemporal Lobar Degeneration
52	Werner Syndrome	113	Crigler-Najjar Syndrome	174	Skin Diseases, Metabolic
53	Xeroderma Pigmentosum	114	Gilbert Disease	175	Wasting Syndrome
54	Glucose Metabolism Disorders	115	Jaundice, Chronic Idiopathic	176	Water-Electrolyte Imbalance
55	Diabetes Mellitus	116	Barth Syndrome	177	Dehydration
56	Diabetes Mellitus, Experimental	117	Hyperlipidemia, Familial Combined	178	Hyperkalemia
57	Diabetes Mellitus, Type 1	118	Hyperlipoproteinemia Type I	179	Hypokalemia
58	Diabetes Mellitus, Type 2	119	Hyperlipoproteinemia Type II	180	Hyponatremia
59	Diabetes, Gestational	120	Hyperlipoproteinemia Type III	181	Water Intoxication
60	Diabetic Ketoacidosis	121	Hyperlipoproteinemia Type V		
61	Donohue Syndrome	122	Lysosomal Storage Dseases		

[Appendix B] Comparison with an Existing Disease Network

To compare the effects of $PPI^{(0)}$ and $PPI^{(0\sim3)}$, we visualized the constructed network with 2,411 diseases that can obtain disease-protein relation information. Figure B1 shows a snapshot of disease network for the proposed network vs. the existing one by Goh et al.'s. Each color of the node represents the disease taxonomy of MeSH, which means the group to which each disease belongs. Note that Goh et al.(2007) is analogous with our PPI(0). In Goh et al.'s approach, there are few edges connecting disease nodes. In contrast, in $\boldsymbol{PPI^{(0\sim3)}}$, all disease nodes are connected, and each has a high degree of node connectivity. The differences in the application of disease scoring to both networks are described in Appendix D.

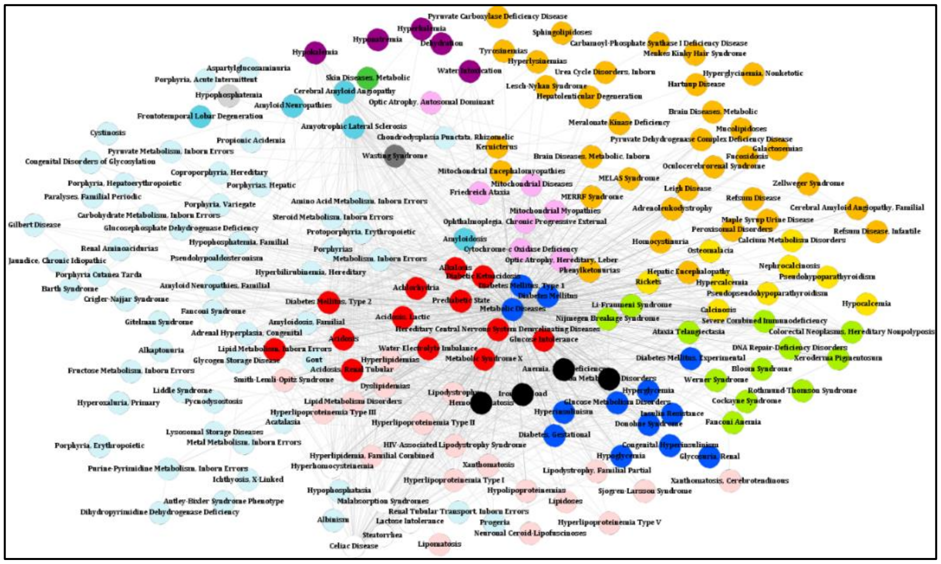

(a) Goh *et al.*'s (2007)

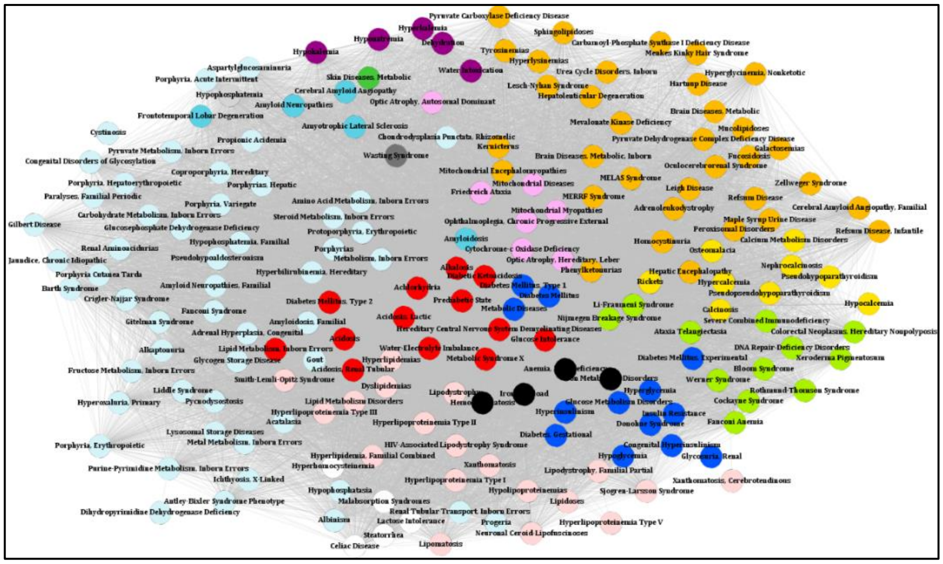

(b) Proposed Disease Network

- Acid-Base Imbalance
- Brain Diseases, Metabolic
- Calcium Metabolism Disorders
- DNA Repair-Deficiency Disorders
- Wasting Syndrome
- Glucose Metabolism Disorders
- Iron Metabolism Disorders
- Lipid Metabolism Disorders
- Malabsorption Syndromes
- Skin Diseases, Metabolic
- Metabolism, Inborn Errors
- Mitochondrial Diseases
- Phosphorus Metabolism Disorders
- Proteostasis Deficiencies
- Water-Electrolyte Imbalance

Figure B1. Comparison an existing disease network of Goh *et al*. (a) and the proposed network (b):

[Appendix C] Table C1. List of Comorbidity Diseases

	Comorbid Diseases	References
1	Acidosis ~ Diabetes Mellitus, Type 2	[C1]
2	Homocystinuria ~ Insulin Resistance	[C2]
3	Liddle Syndrome ~ Pseudohypoaldosteronism	[C3]
4	Rickets ~ Malabsorption Syndromes	[C4]
5	Rickets ~ Hypophosphatasia	[C5]
6	Maple Syrup Urine Disease ~ Phenylketonurias	[C6]
7	Hyperglycemia ~ Insulin Resistance	[C7]
8	Hyperlipoproteinemia Type III ~ Albinism	[C8]
9	Hyperlipoproteinemia Type II ~ Gout	[C9]
10	Diabetes Mellitus, Type 2 ~ Hyperlipoproteinemia Type III	[C10]
11	Insulin Resistance ~ Ataxia Telangiectasia	[C11]
12	Homocystinuria ~ Metabolism, Inborn Errors	[C12]
13	Hyperhomocysteinemia ~ Homocystinuria	[C13]
14	Diabetes Mellitus, Type 2 ~ Congenital Hyperinsulinism	[C14]
15	Hypoglycemia ~ Congenital Hyperinsulinism	[C15,C16,C17]
16	Albinism ~ Maple Syrup Urine Disease	[C17]
17	Maple Syrup Urine Disease ~ Metabolism, Inborn Errors	[C18]
18	Acidosis, Lactic ~ Pyruvate Carboxylase Deficiency Disease	[C19]
19	Leigh Disease ~ Pyruvate Dehydrogenase Complex Deficiency Disease	[C20, C59]
20	Acidosis, Lactic ~ Pyruvate Dehydrogenase Complex Deficiency Disease	[C21]
21	Acidosis, Lactic ~ Mitochondrial Encephalomyopathies	[C22]
22	Acidosis, Lactic ~ Diabetes Mellitus, Type 2	[C23]
23	Acidosis, Lactic ~ Diabetic Ketoacidosis	[C24]
24	Ataxia Telangiectasia ~ Nijmegen Breakage Syndrome	[C25]
25	Dyslipidemias ~ Amyotrophic Lateral Sclerosis	[C26]
26	Hypocalcemia ~ Pseudohypoparathyroidism	[C27,C71,C72]
27	Pseudohypoparathyroidism ~ Diabetes Mellitus, Type 1	[C28]
28	Hemochromatosis ~ Iron Overload	[C29,C35]
29	Alkaptonuria ~ Metabolism, Inborn Errors	[C30]
30	Amyloidosis ~ Cerebral Amyloid Angiopathy	[C31]
31	Hyperoxaluria, Primary ~ Steatorrhea	[C32]
32	Fanconi Syndrome ~ Cystinosis	[C33]
33	Hypoglycemia ~ Diabetes Mellitus, Type 2	[C34,C44]
34	Fanconi Syndrome ~ Osteomalacia	[C36]
35	Diabetic Ketoacidosis ~ Diabetes Mellitus, Type 2	[C37,C64]
36	Hyperlipidemias ~ Diabetic Ketoacidosis	[C38]
37	Diabetes Mellitus, Type 2 ~ Dyslipidemias	[C39,CA49,C50,C67]
38	Dyslipidemias ~ Metabolic Syndrome X	[C40,C51]
39	Hyperinsulinism ~ Insulin Resistance	[C41,C61]
40	Diabetes Mellitus, Type 2 ~ Metabolic Syndrome X	[C42,C60,C74]
41	Insulin Resistance ~ Metabolic Syndrome X	[C43,C51]
42	Insulin Resistance ~ Diabetes Mellitus, Type 2	[C45,C49,C61]
43	Dyslipidemias ~ Insulin Resistance	[C43,C46,C47,C48,C49,C51,C52]
44	Hyperglycemia ~ Hyperlipidemias	[C46]
45	Hyperinsulinism ~ Hypoglycemia	[C53]
46	Hyperlipoproteinemia Type V ~ Diabetes Mellitus, Type 2	[C54]
47	Acidosis, Renal Tubular ~ Fanconi Syndrome	[C55,C58]
48	Insulin Resistance ~ Werner Syndrome	[C56]
49	Acidosis, Lactic ~ Fanconi Syndrome	[C57]
50	Diabetes Mellitus ~ Hyperglycemia	[C63]
51	Diabetic Ketoacidosis ~ Diabetes Mellitus, Type 1	[C64,C65]
52	Diabetic Ketoacidosis ~ Hyperglycemia	[C66]
53	Hypoglycemia ~ Diabetic Ketoacidosis	[C68]
54	Acidosis, Lactic ~ Melas Syndrome	[C69,C70]
55	Metabolic Syndrome X ~ Skin Diseases, Metabolic	[C73]
56	Skin Diseases, Metabolic ~ Diabetes Mellitus, Type 2	[C74,C75]
57	Amyloidosis, Familial ~ Alkaptonuria	[C76]
58	Liddle Syndrome ~ Alkalosis	[C77]
59	Diabetes Mellitus, Type 1 ~ Diabetes Mellitus, Type 2	[C64]
60	Alkalosis ~ Hypokalemia	[C77]
61	Liddle Syndrome ~ Hypokalemia	[C77]
62	Hyperinsulinism ~ Diabetes Mellitus, Type 2	[C41,C62]

References for Comorbidity Analysis

[C1] Bodmer M, Meier C, Krähenbühl S, Jick SS, Meier CR: Metformin, Sulfonylureas, or Other Antidiabetes Drugs and the Risk of Lactic Acidosis or Hypoglycemia A nested case-control analysis. *Diabetes Care* 2008, 31(11):2086-2091.

[C2] Stagi S, Lapi E, Pantaleo M, Chiarelli F, Seminara S, De Martino M: Type II diabetes and impaired glucose tolerance due to severe hyperinsulinism in patients with 1p36 deletion syndrome and a Prader-Willi-like phenotype. *BMC medical genetics* 2014, 15(1):16.

[C3] Schild L: The ENaC channel as the primary determinant of two human diseases: Liddle syndrome and pseudohypoaldosteronism. *Nephrologie* 1995, 17(7):395-400.

[C4] Minambres I, Chico A, Perez A: Severe hypocalcemia due to vitamin D deficiency after extended Roux-en-Y gastric bypass. *Journal of obesity*, 2011.

[C5] Ma H: Rickets-like genetic diseases. *Zhongguo dang dai er ke za zhi= Chinese journal of contemporary pediatrics* 2013, 15(11):923-927.

[C6] Walterfang M, Bonnot O, Mocellin R, Velakoulis D: The neuropsychiatry of inborn errors of metabolism. *Journal of inherited metabolic disease* 2013, 36(4):687-702.

[C7] Ryo M, Furiya Y, Ueno S: Metabolic syndrome. *Nihon rinsho Japanese journal of clinical medicine* 2014, 72(4):702-707.

[C8] Sobra J, Jílek M: Inborn defects of lipid metabolism. 23. Coincidence of type 3 hyperlipoproteinemia and albinism. *Casopis lekaru ceskych* 1971, 110(19):451-453.

[C9] Jiao S, Kameda K, Matsuzawa Y, Tarui S: Hyperlipoproteinaemia in primary gout: hyperlipoproteinaemic phenotype and influence of alcohol intake and obesity in Japan. *Annals of the rheumatic diseases* 1986, 45(4):308-313.

[C10] Eto M, Saito M: Familial type III hyperlipoproteinemia. *Nihon rinsho Japanese journal of clinical medicine* 2013, 71(9):1590-1594.

[C11] Bar RS, Levis WR, Rechler MM, Harrison LC, Siebert C, Podskalny J, Roth J, Muggeo M: Extreme insulin resistance in ataxia telangiectasia: defect in affinity of insulin receptors. *New England Journal of Medicine* 1978, 298(21):1164-1171.

[C12] Wada M, Kuroki M, Minami Y, Ikeda R, Sekitani Y, Takamura N, Kawakami S, Kuroda N, Nakashima K: Quantitation of sulfur-containing amino acids, homocysteine, methionine and cysteine in dried blood spot from newborn baby by HPLC-fluorescence detection. *Biomedical Chromatography* 2014, 28(6):810-814.

[C13] Poloni S, Schweigert PI, D'Almeida V, Schwartz I: Does phase angle correlate with hyperhomocysteinemia? A study of patients with classical homocystinuria. *Clinical nutrition (Edinburgh, Scotland)* 2013, 32(3):479-480.

[C14] Tornovsky-Babeay S, Dadon D, Ziv O, Tzipilevich E, Kadosh T, Schyr-Ben Haroush R, Hija A, Stolovich-Rain M, Furth-Lavi J, Granot Z: Type 2 Diabetes and Congenital Hyperinsulinism Cause DNA Double-Strand Breaks and p53 Activity in β Cells. *Cell metabolism* 2014, 19(1):109-121.

[C15] Neylon OM, Moran MM, Pellicano A, Nightingale M, O'Connell MA: Successful subcutaneous glucagon use for persistent hypoglycaemia in congenital hyperinsulinism. *Journal of Pediatric Endocrinology and Metabolism* 2013, 26(11-12):1157-1161.

[C16] Dillon PA: Congenital hyperinsulinism. *Current opinion in pediatrics* 2013, 25(3):357-361.

[C17] Nagabushana D, Benakappa A: Maple syrup urine disease and oculocutaneous albinism in twins. *Journal of clinical neonatology* 2014, 3(1):55.

[C18] Mescka CP, Wayhs CAY, Vanzin CS, Biancini GB, Guerreiro G, Manfredini V, Souza C, Wajner M, Dutra-filho CS, Vargas CR: Protein and lipid damage in maple syrup urine disease patients: l-carnitine effect. *International Journal of Developmental Neuroscience* 2013, 31(1):21-24.

[C19] García-Cazorla A, Rabier D, Touati G, Chadefaux-Vekemans B, Marsac C, de Lonlay P, Saudubray JM: Pyruvate carboxylase deficiency: metabolic characteristics and new neurological aspects. *Annals of neurology* 2006, 59(1):121-127.

[C20] Hinman L, Sheu KR, Baker A, Kim Y, Blass J: Deficiency of pyruvate dehydrogenase complex (PDHC) in Leigh's disease fibroblasts An abnormality in lipoamide dehydrogenase affecting PDHC activation. *Neurology* 1989, 39(1):70-70.

[C21] Ferriero R, Manco G, Lamantea E, Nusco E, Ferrante MI, Sordino P, Stacpoole PW, Lee B, Zeviani M, Brunetti-Pierri N: Phenylbutyrate therapy for pyruvate dehydrogenase complex deficiency and lactic acidosis. *Science translational medicine* 2013, 5(175):175ra131-175ra131.

[C22] Luft FC: Lactic acidosis update for critical care clinicians. *Journal of the American Society of Nephrology* 2001, 12(suppl 1):S15-S19.

[C23] Fimognari FL, Pastorelli R, Incalzi RA: Phenformin-Induced Lactic Acidosis in an Older Diabetic Patient A recurrent drama (phenformin and lactic acidosis). *Diabetes care* 2006, 29(4):950-951.

[C24] Feenstra RA, Kiewiet MK, Boerma EC, ter Avest E: Lactic acidosis in diabetic ketoacidosis. *BMJ case reports* 2014, 2014:bcr2014203594.

[C25] Bienemann K, Burkhardt B, Modlich S, Meyer U, Möricke A, Bienemann K, Mauz-Körholz C, Escherich G, Zimmermann M, Körholz D: Promising therapy results for lymphoid malignancies in children with chromosomal breakage syndromes (Ataxia teleangiectasia or Nijmegen-breakage syndrome): a retrospective survey. *British journal of haematology* 2011, 155(4):468-476.

[C26] Dupuis L, Corcia P, Fergani A, De Aguilar J-LG, Bonnefont-Rousselot D, Bittar R, Seilhean D, Hauw J-J, Lacomblez L, Loeffler J-P: Dyslipidemia is a protective factor in amyotrophic lateral sclerosis. *Neurology* 2008, 70(13):1004-1009.

[C27] Dosi RV, Ambaliya AP, Joshi HK, Patell RD: Pseudohypoparathyroidism, Rare Cause of Hypocalcaemia! *Journal of clinical and diagnostic research: JCDR* 2013, 7(10):2288.

[C28] Saikia B, Arora S, Puliyel JM: Pseudohypoparathyroidism with diabetes mellitus and hypothyroidism. *Indian pediatrics* 2012, 49(12):989-991.

[C29] McLaren CE, Gordeuk VR, Chen W-P, Barton JC, Acton RT, Speechley M, Castro O, Adams PC, Snively BM, Harris EL: Bivariate mixture modeling of transferrin saturation and serum ferritin concentration in Asians, African Americans, Hispanics, and whites in the Hemochromatosis and Iron Overload Screening (HEIRS) Study. *Translational Research* 2008, 151(2):97-109.
[C30] Aquaron RR: Alkaptonuria in France: past experience and lessons for the future. *Journal of inherited metabolic disease* 2011, 34(6):1115-1126.
[C31] Ghiso J, Fossati S, Rostagno A: Amyloidosis Associated with Cerebral Amyloid Angiopathy: Cell Signaling Pathways Elicited in Cerebral Endothelial Cells. *Journal of Alzheimer's Disease* 2014.
[C32] Canales BK, Ellen J, Khan SR, Hatch M: Steatorrhea and hyperoxaluria occur after gastric bypass surgery in obese rats regardless of dietary fat or oxalate. *The Journal of urology* 2013, 190(3):1102-1109.
[C33] Besouw M, Cornelissen E, Cassiman D, Kluijtmans L, van den Heuvel L, Levtchenko E: Carnitine Profile and Effect of Suppletion in Children with Renal Fanconi Syndrome due to Cystinosis. 2014.
[C34] Lopez JM, Annunziata K, Bailey RA, Rupnow MF, Morisky DE: Impact of hypoglycemia on patients with type 2 diabetes mellitus and their quality of life, work productivity, and medication adherence. *Patient preference and adherence* 2014, 8:683.
[C35] Abu Rajab M, Guerin L, Lee P, Brown KE: Iron overload secondary to cirrhosis: a mimic of hereditary haemochromatosis? *Histopathology* 2014.
[C36] Terasaka T, Ueta E, Ebara H, Waseda K, Hanayama Y, Takaki A, Kawabata T, Sugiyama H, Hidani K, Otsuka F: Long-term Observation of Osteomalacia Caused by Adefovir-Induced Fanconi's Syndrome. *Acta Medica Okayama* 2014, 68(1):53-56.
[C37] Umpierrez GE, Smiley D, Kitabchi AE: Narrative review: ketosis-prone type 2 diabetes mellitus. *Annals of internal medicine* 2006, 144(5):350-357.
[C38] Williamson S, Alexander V, Greene SA: Severe hyperlipidaemia complicating diabetic ketoacidosis. *Archives of disease in childhood* 2012, 97(8):735-735.
[C39] Yuan C, Lai CW, Chan LW, Chow M, Law HK, Ying M: Cumulative Effects of Hypertension, Dyslipidemia, and Chronic Kidney Disease on Carotid Atherosclerosis in Chinese Patients with Type 2 Diabetes Mellitus. *Journal of diabetes research* 2014, 2014.
[C40] Shin JA, Lee JH, Lim SY, Ha HS, Kwon HS, Park YM, Lee WC, Kang MI, Yim HW, Yoon KH: Metabolic syndrome as a predictor of type 2 diabetes, and its clinical interpretations and usefulness. *Journal of diabetes investigation* 2013, 4(4):334-343.
[C41] Shalimova A: Correction of endothelial dysfunction in patients with essential hypertension and type 2 diabetes. *Georgian medical news* 2014(229):33-40.
[C42] Gragnoli C: Hypothesis of the neuroendocrine cortisol pathway gene role in the comorbidity of depression, type 2 diabetes, and metabolic syndrome. *The application of clinical genetics* 2014, 7:43.
[C43] Farr S, Taher J, Adeli K: Glucagon-Like Peptide-1 as a Key Regulator of Lipid and Lipoprotein Metabolism in Fasting and Postprandial States. *Cardiovascular & hematological disorders drug targets* 2014.
[C44] Pilotto A, Noale M, Maggi S, Addante F, Tiengo A, Perin PC, Rengo G, Crepaldi G: Hypoglycemia Is Independently Associated with Multidimensional Impairment in Elderly Diabetic Patients. *BioMed research international* 2014, 2014.
[C45] Belovol A, Shalimova A, Kochueva M: Structural and functional changes of heart and vessels in patients with essential hypertension and type 2 diabetes. *Georgian medical news* 2014(228):45-51.
[C46] Farr S, Adeli K: Incretin-based therapies for treatment of postprandial dyslipidemia in insulin-resistant states. *Current opinion in lipidology* 2012, 23(1):56-61.
[C47] Hsieh J, Hayashi AA, Webb J, Adeli K: Postprandial dyslipidemia in insulin resistance: mechanisms and role of intestinal insulin sensitivity. *Atherosclerosis Supplements* 2008, 9(2):7-13.
[C48] Avramoglu RK, Qiu W, Adeli K: Mechanisms of metabolic dyslipidemia in insulin resistant states: deregulation of hepatic and intestinal lipoprotein secretion. *Frontiers in bioscience: a journal and virtual library* 2003, 8:d464-476.
[C49] Grigoropoulou P, Eleftheriadou I, Zoupas C, Diamanti-Kandarakis E, Tentolouris N: Incretin-based Therapies for Type 2 Diabetes Mellitus: Effects on Insulin Resistance. *Current diabetes reviews* 2013, 9(5):412-417.
[C50] Klop B, Elte JWF, Cabezas MC: Dyslipidemia in obesity: mechanisms and potential targets. *Nutrients* 2013, 5(4):1218-1240.
[C51] Holvoet P: Relations between metabolic syndrome, oxidative stress and inflammation and cardiovascular disease. *Verhandelingen-Koninklijke Academie voor Geneeskunde van Belgie* 2007, 70(3):193-219.
[C52] Reaven G: Insulin resistance, the insulin resistance syndrome, and cardiovascular disease. *Panminerva medica* 2005, 47(4):201-210.
[C53] Weinzimer SA, Stanley CA, Berry GT, Yudkoff M, Tuchman M, Thornton PS: A syndrome of congenital hyperinsulinism and hyperammonemia. *The Journal of pediatrics* 1997, 130(4):661-664.
[C54] Park JR, Jung TS, Jung JH, Lee G-W, Kim M, Park K-J, Kim DR, Chang S-H, Chung SI, Hahm JR: A case of hypothyroidism and type 2 diabetes associated with type V hyperlipoproteinemia and eruptive xanthomas. *Journal of Korean medical science* 2005, 20(3):502-505.
[C55] Sebastian A, McSherry E, Morris Jr RC: On the mechanism of renal potassium wasting in renal tubular acidosis associated with the Fanconi syndrome (type 2 RTA). *Journal of Clinical Investigation* 1971, 50(1):231.
[C56] Donadille B, D'Anella P, Auclair M, Uhrhammer N, Sorel M, Grigorescu R, Ouzounian S, Cambonie G, Boulot P, Laforêt P: Partial lipodystrophy with severe insulin resistance and adult progeria Werner syndrome. *Orphanet journal of rare diseases* 2013, 8(1):106.
[C57] Nelson M, Azwa A, Sokwala A, Harania RS, Stebbing J: Fanconi syndrome and lactic acidosis associated with stavudine and lamivudine therapy. *Aids* 2008, 22(11):1374-1376.

[C58] Taylor HC, Elbadawy EH: Renal tubular acidosis type 2 with Fanconi's syndrome, osteomalacia, osteoporosis, and secondary hyperaldosteronism in an adult consequent to vitamin D and calcium deficiency: effect of vitamin D and calcium citrate therapy. *Endocrine practice* 2006, 12(5):559-567.

[C59] Kretzschmar HA, DeArmond SJ, Koch TK, Patel MS, Newth CJ, Schmidt KA, Packman S: Pyruvate dehydrogenase complex deficiency as a cause of subacute necrotizing encephalopathy (Leigh disease). *Pediatrics* 1987, 79(3):370-373.

[C60] Boney CM, Verma A, Tucker R, Vohr BR: Metabolic syndrome in childhood: association with birth weight, maternal obesity, and gestational diabetes mellitus. *Pediatrics* 2005, 115(3):e290-e296.

[C61] Zimmet P, Boyko E, Collier G, Courten Md: Etiology of the metabolic syndrome: potential role of insulin resistance, leptin resistance, and other players. *Annals of the New York Academy of Sciences* 1999, 892(1):25-44.

[C62] Luchsinger JA: Diabetes, related conditions, and dementia. *Journal of the neurological sciences* 2010, 299(1):35-38.

[C63] Sakurai T: Preventive strategy for cognitive decline in elderly with diabetes mellitus. *Nihon rinsho Japanese journal of clinical medicine* 2014, 72(4):692-696.

[C64] Barski L, Nevzorov R, Harman-Boehm I, Jotkowitz A, Rabaev E, Zektser M, Zeller L, Shleyfer E, Almog Y: Comparison of diabetic ketoacidosis in patients with type-1 and type-2 diabetes mellitus. *The American journal of the medical sciences* 2013, 345(4):326-330.

[C65] White NH: Diabetic ketoacidosis in children. *Endocrinology and metabolism clinics of North America* 2000, 29(4):657-682.

[C66] Rewers A: Current concepts and controversies in prevention and treatment of diabetic ketoacidosis in children. *Current diabetes reports* 2012, 12(5):524-532.

[C67] Shah S, Arneja J: Efficacy of rosuvastatin in achieving target HDL, LDL, triglycerides and total cholesterol levels in type 2 diabetes mellitus (T2DM) with newly diagnosed dyslipidaemia: an open label, nonrandomised, non-interventional and observational study in India. *The Journal of the Association of Physicians of India* 2013, 61(10):721-726, 732.

[C68] Sharda S, Angurana SK, Walia M, Attri S: Defect of Cobalamin Intracellular Metabolism Presenting as Diabetic Ketoacidosis: A Rare Manifestation. In: *JIMD Reports-Volume 11*. Springer; 2013: 43-47.

[C69] Leng Y, Liu Y, Fang X, Li Y, Yu L, Yuan Y, Wang Z: The mitochondrial DNA 10197 G> A mutation causes MELAS/Leigh overlap syndrome presenting with acute auditory agnosia. *Mitochondrial DNA* 2014(0):1-5.

[C70] Santa KM: Treatment Options for Mitochondrial Myopathy, Encephalopathy, Lactic Acidosis, and Stroke-Like Episodes (MELAS) Syndrome. *Pharmacotherapy: The Journal of Human Pharmacology and Drug Therapy* 2010, 30(11):1179-1196.

[C71] Wang O, Xing X-p, Meng X-w, Xia W-b, Li M, Jiang Y, HU Y-Y, LIU H-C: Treatment of hypocalcemia caused by hypoparathyroidism or pseudohypoparathyroidism with domestic-made calcitriol: a prospective and self-controlled clinical trial. *Chinese Medical Journal (English Edition)* 2009, 122(3):279.

[C72] Cho MJ, Ban KH, Park JA, Lee HD: Congestive Heart Failure: An Unusual Presentation of Pseudohypoparathyroidism. *Pediatric emergency care* 2013, 29(7):826-828.

[C73] Wolska K, Michalska-Jakubus M, Pietrzak A, Krasowska D: Metabolic syndrome in patients with psoriasis. *Polski merkuriusz lekarski: organ Polskiego Towarzystwa Lekarskiego* 2014, 36(213):215-219.

[C74] Wilson P, Bohjanen K, Ingraham S, Leon A: Psoriasis and physical activity: a review. *Journal of the European Academy of Dermatology and Venereology* 2012, 26(11):1345-1353.

[C75] Davidovici BB, Sattar N, Jörg PC, Puig L, Emery P, Barker JN, van de Kerkhof P, Ståhle M, Nestle FO, Girolomoni G: Psoriasis and systemic inflammatory diseases: potential mechanistic links between skin disease and co-morbid conditions. *Journal of Investigative Dermatology* 2010, 130(7):1785-1796.

[C76] Millucci L, Ghezzi L, Paccagnini E, Giorgetti G, Viti C, Braconi D, Laschi M, Geminiani M, Soldani P, Lupetti P: Amyloidosis, Inflammation, and Oxidative Stress in the Heart of an Alkaptonuric Patient. *Mediators of inflammation* 2014, 2014.

[C77] Ferraro F, Meschi M, Detrenis S, Savazzi G: Differential diagnosis in hypokalemia. A case of Liddle syndrome. *Recenti progressi in medicina* 2004, 95(2):87-95.

[Appendix D] Performance comparison with single network and integrated network

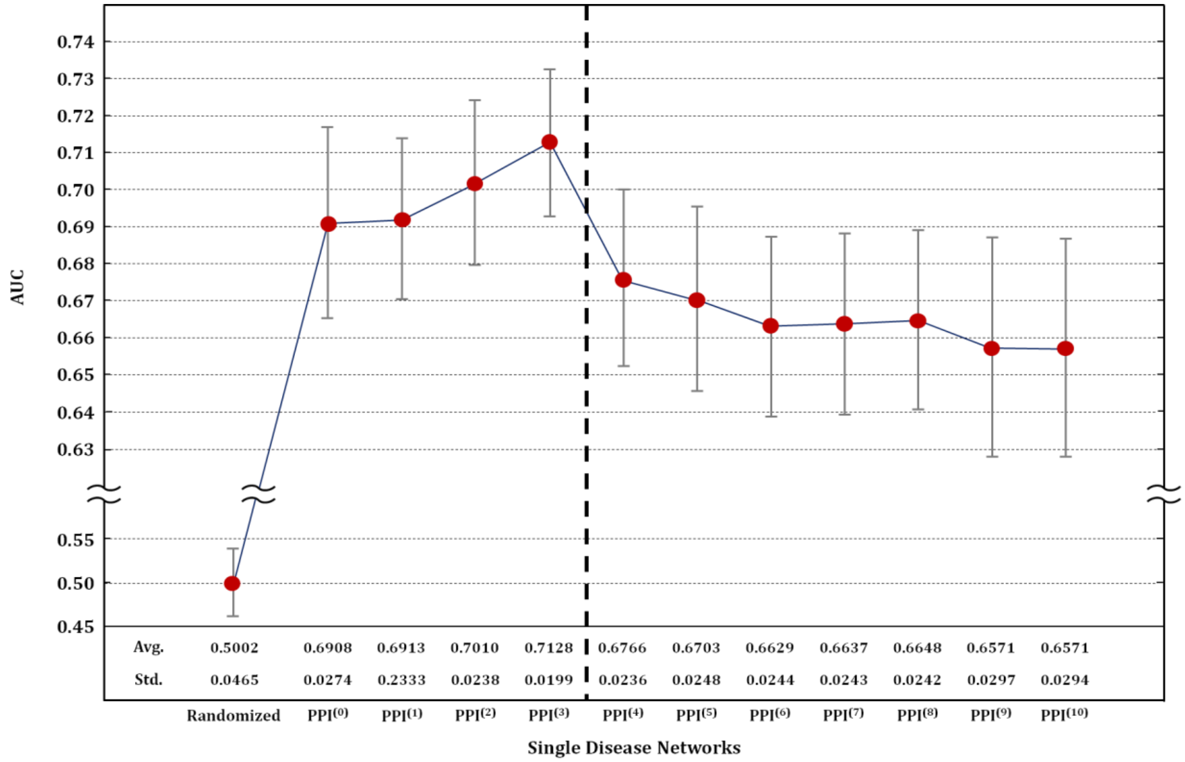

Figure D1. **AUC comparison from $q = 0$ to 10 for individual networks:** The average AUC value with the standard deviation is presented as a circle with an error bar. The AUCs of individual disease networks increase up to $q = 3$, but after then, it begins to decrease. It is conjecture that decrease in network connectivity negatively affects to performance.

Table D1. **AUC comparison from $q = 0$ to 10 for integrated networks:** the *p*-values are obtained from pairwise comparisons, $\text{PPI}^{(0\sim3)}$ vs. $\text{PPI}^{(0\sim q)}$

Network	Avg.	Std.	*p*-value	Network	Avg.	Std.	*p*-value
**Randomized**	0.5002	0.0465	1.01×10^{-65}	**$\text{PPI}^{(0\sim5)}$**	0.7127	0.0182	3.48×10^{-6}
**$\text{PPI}^{(0)}$**	0.6908	0.0274	7.85×10^{-13}	**$\text{PPI}^{(0\sim6)}$**	0.7180	0.0208	5.94×10^{-7}
**$\text{PPI}^{(0\sim1)}$**	0.6999	0.0274	9.39×10^{-10}	**$\text{PPI}^{(0\sim7)}$**	0.7110	0.0232	7.13×10^{-6}
**$\text{PPI}^{(0\sim2)}$**	0.7021	0.0241	4.89×10^{-7}	**$\text{PPI}^{(0\sim8)}$**	0.7038	0.0251	1.02×10^{-2}
**$\text{PPI}^{(0\sim3)}$**	0.7170	0.0176	–	**$\text{PPI}^{(0\sim9)}$**	0.6997	0.0252	3.21×10^{-3}
**$\text{PPI}^{(0\sim4)}$**	0.7186	0.0207	2.47×10^{-6}	**$\text{PPI}^{(0\sim10)}$**	0.6947	0.0259	2.97×10^{-4}

[Appendix E] Comparative results for scoring

Scoring experiments were performed on three disease groups; metabolic diseases, neoplasms, and nervous system diseases. by setting the label of a 20% disease on '$\boldsymbol{y_l} = \mathbf{1}$', while keeping unchanged the labels of the remaining 80% diseases as '$\boldsymbol{y_u} = \mathbf{0}$', we obtained the predicted score for identifying the comorbid diseases with the given disease. Five sets of experiments per disease group were carried out in a similar manner for 5-fold cross-validation, and this procedure was repeated 10 times. the performance was measured by AUC (the area under the receiver operating characteristic curve). $PPI^{(0)}$ is a scoring result using Goh *et al*'s network, and $PPI^{(0\sim3)}$ is a translational network proposed in this study. Also, we provide preliminary scoring results using diseases belonging to Neoplasms and Nervous System diseases (See, Appendix H). Integrated network means piling-up networks with $PPI^{(0)}$ up to $PPI^{(q)}$, q = 1,2,3.

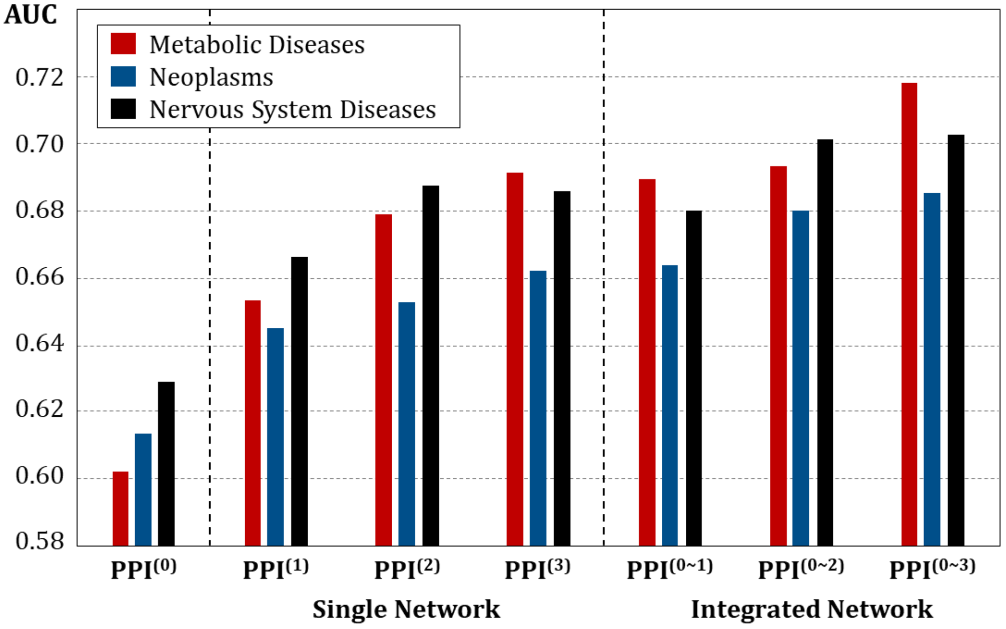

Figure E1. Comparative results for scoring with single and integrated network

[Appendix F] More Examples on Associated Diseases by Scoring Results.

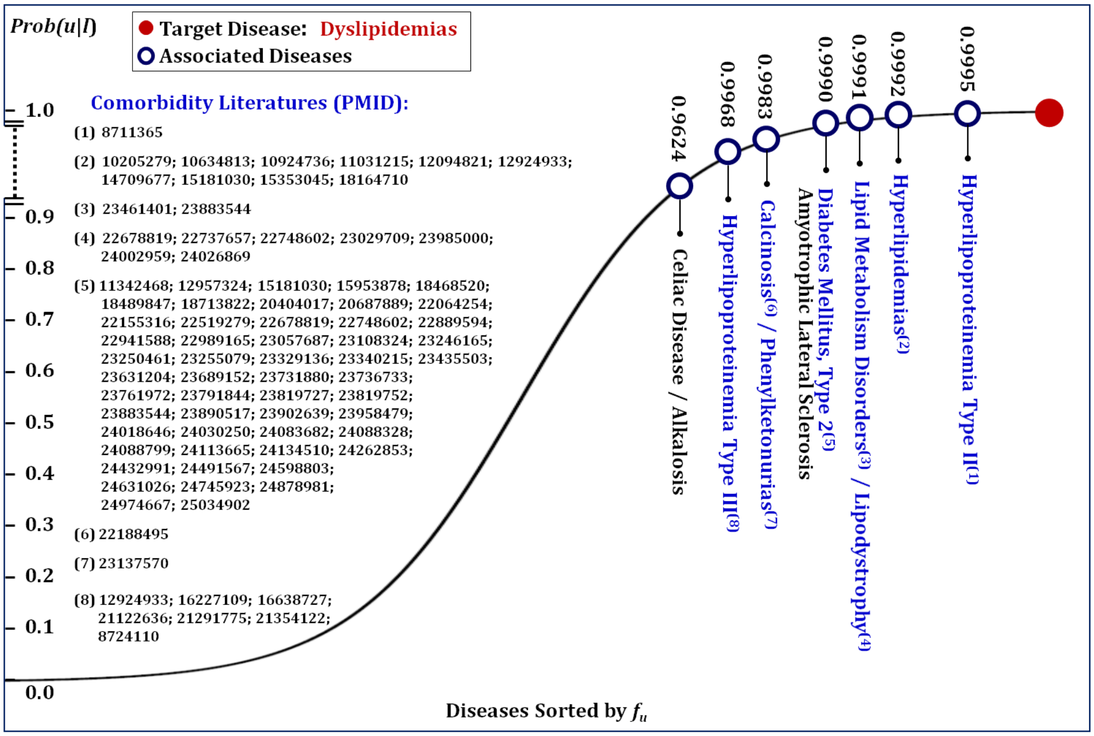

Probabilities of the diseases associated with Dyslipidemias

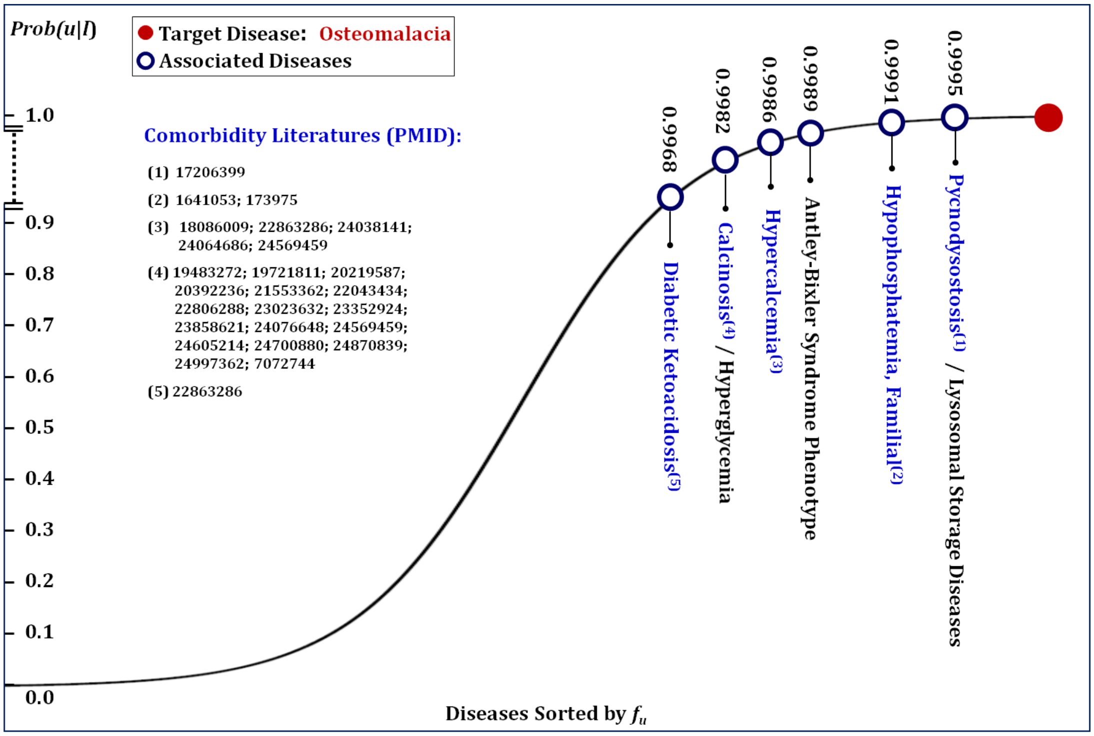

Probabilities of the diseases associated with Osteomalacia

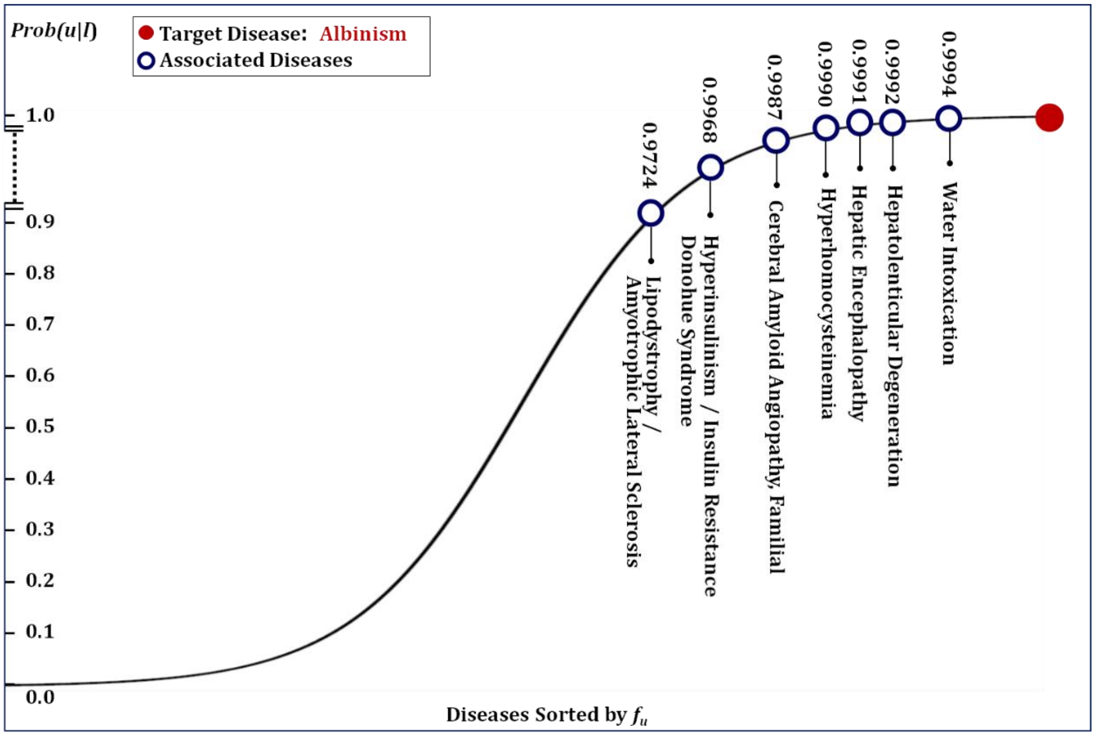

Probabilities of the diseases associated with Albinism

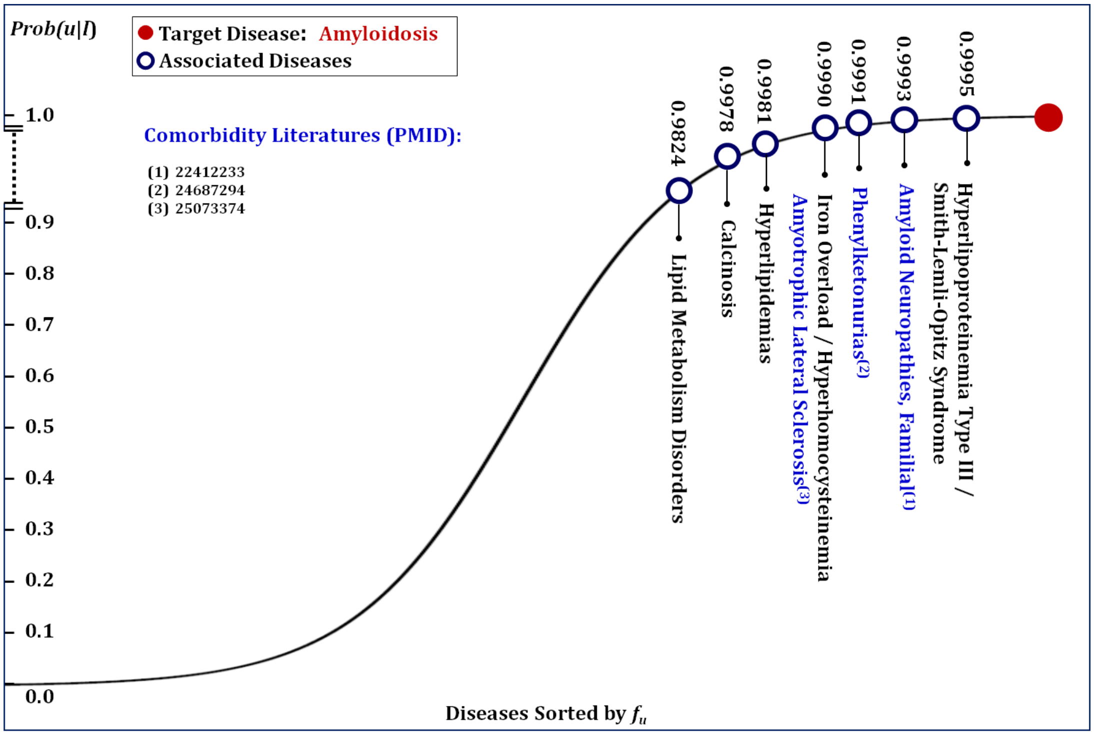

Probabilities of the diseases associated with Amyloidosis

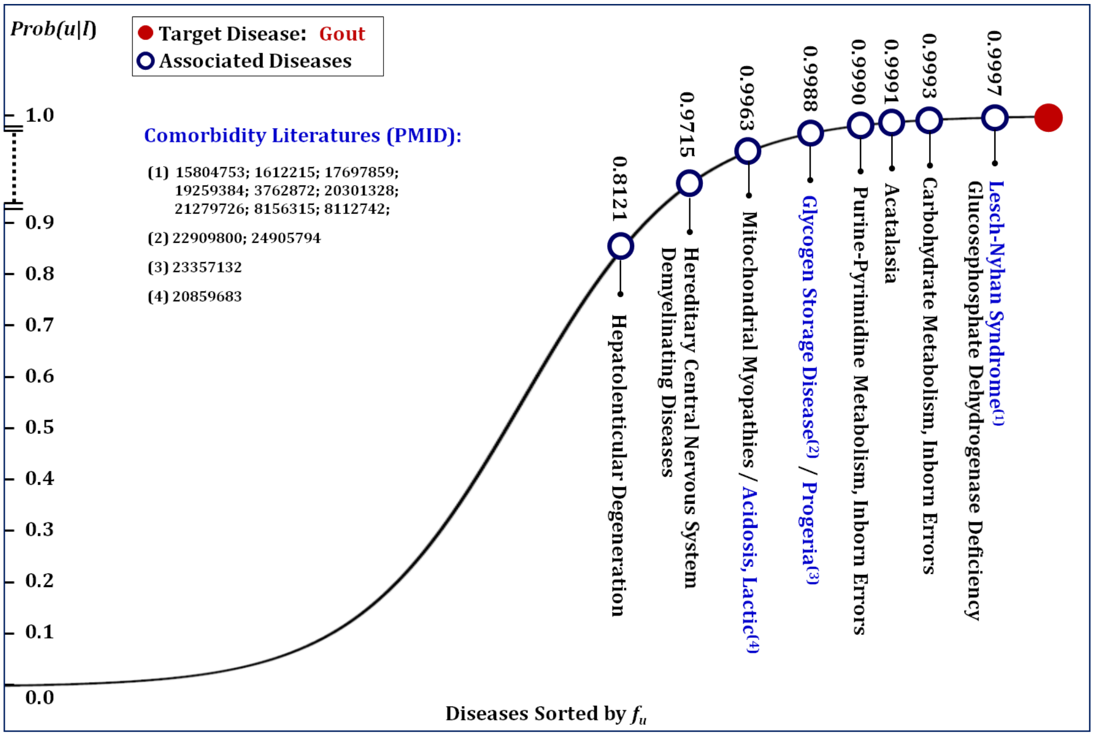

Probabilities of the diseases associated with Gout

[Appendix G] Connected Genes and Diseases of Gout

The following figure shows a typical case about how a disease is linked to other diseases, and therein, which gene turns out to be important (essential) for the connection. Red circle represents target diseases and hexagon represents disease-related genes as q varies. The solid line shows interactions between disease and proteins or between protein and protein.

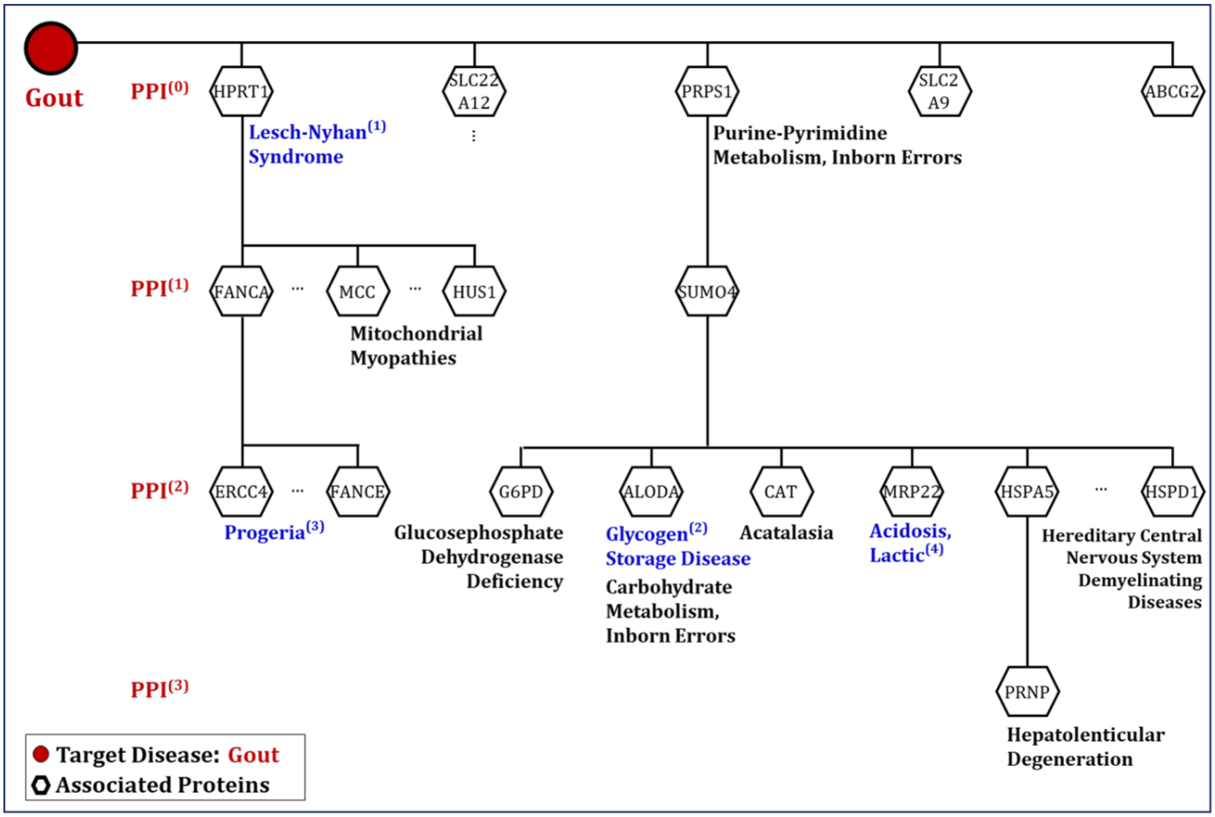

Protein Links for the Associated Diseases with the Target Disease: Gout

[Appendix H] Preliminary Results of Scoring for Other Target Diseases

- **Case for Nervous System Diseases: Alzheimer**

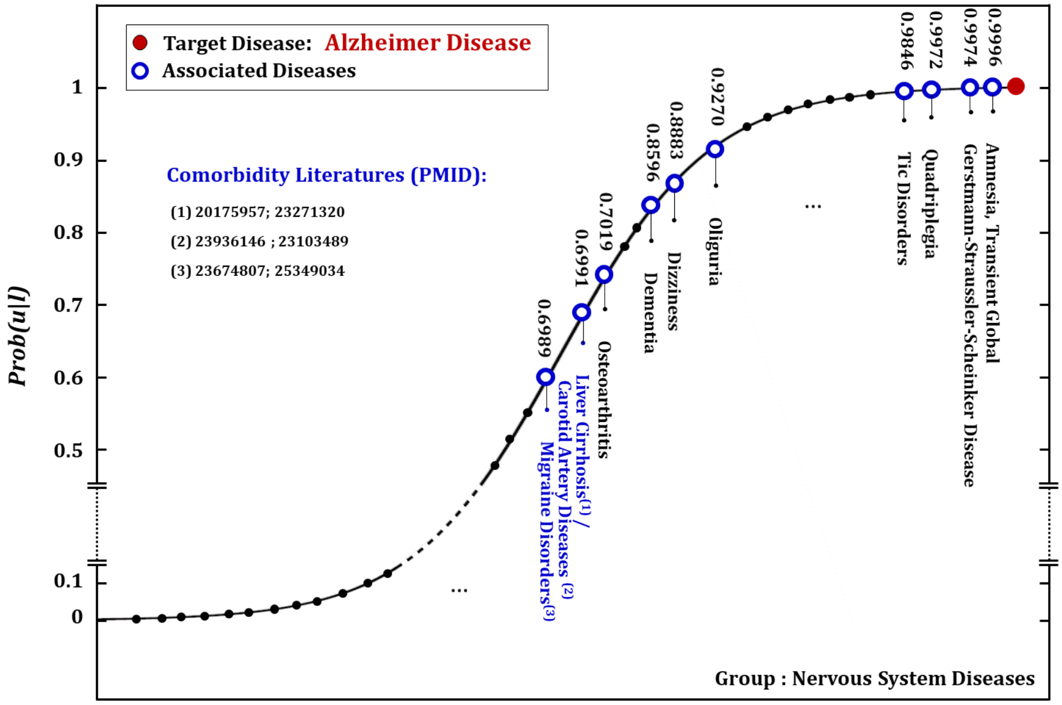

Probabilities of the disease associated with Alzheimer Disease

- **Case for Neoplasms: Breast Neoplasms**

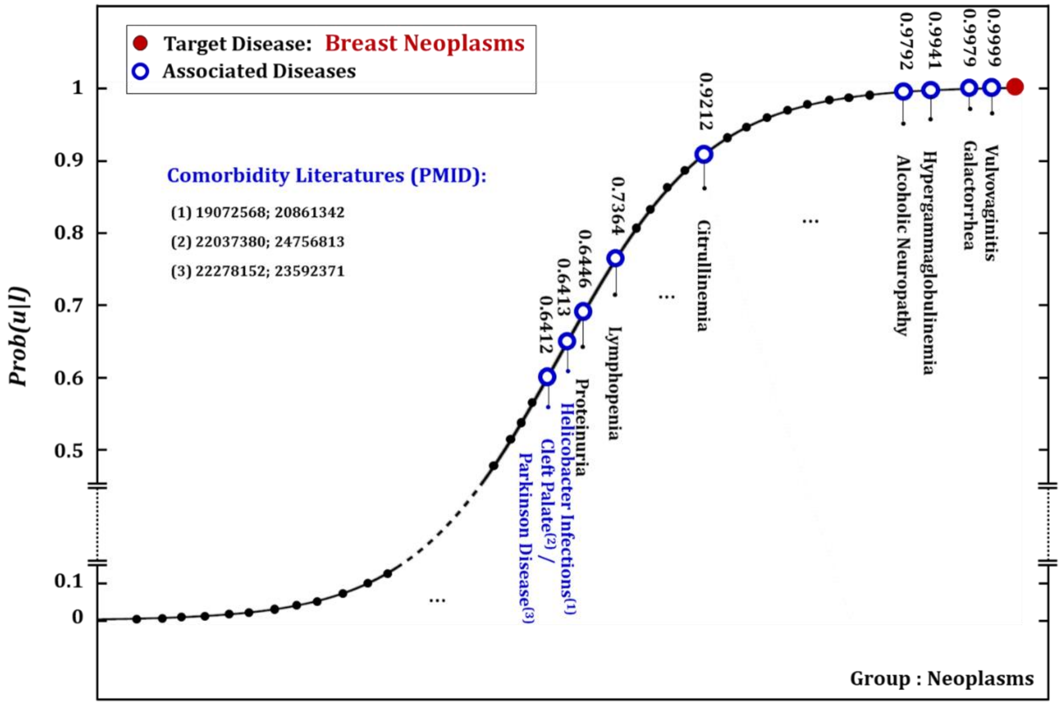

Probabilities of the disease associated with Breast Neoplasms

[Appendix I] Constructing Disease Network by q-step walk on PPI network

Disease networks are constructed based on the notion of *walk* from graph theory on the PPI network. On a graph (or network) $\boldsymbol{G}$, a '*walk (or path)*' starting at node $\boldsymbol{v_A}$ and ending at node $\boldsymbol{v_B}$, is represented as $(\boldsymbol{v_A} \rightarrow \boldsymbol{v_1} \rightarrow \cdots \rightarrow \boldsymbol{v_n} \rightarrow \boldsymbol{v_B})$. The edges connect the successive nodes in a walk. Let us define a '$\boldsymbol{q}$*-step walk'* as a walk of length $\boldsymbol{q}$, which travels $\boldsymbol{q}$ edges departing from $\boldsymbol{v_A}$ for $\boldsymbol{v_B}$. Applying $\boldsymbol{q}$-step walk to the PPI network, denoted as $\boldsymbol{PPI^{(q)}}$, disease-disease relations can be differently defined depending on $\boldsymbol{q}$. The following section lists the proposed disease networks $\boldsymbol{PPI^{(q)}}$*'s* where $\boldsymbol{q} = \boldsymbol{0}, \boldsymbol{1}, \boldsymbol{2}, \boldsymbol{3}$.

- $\boldsymbol{PPI^{(0)}}$: Consider two diseases, *Disease I* and *Disease II*, assuming that the former is known to be associated with protein $\boldsymbol{P_1}$ ($\ni$ *Disease I*) and the latter with $\boldsymbol{P_2}$ ($\ni$ *Disease II*) with respect to the disease-protein relationship [20]. In a conventional approach for constructing a disease network [20], the two diseases are defined as *related* or *associated* ('*Disease I ~ Disease II'* where '~' stands for association) only if $\boldsymbol{P_1}$ and $\boldsymbol{P_2}$ are identical ($\boldsymbol{P_1} \equiv \boldsymbol{P_2}$). This implies there is no edge (*0-step walk*) between the pair on the PPI network. Let us denote this type of disease network as $\boldsymbol{PPI^{(0)}}$. See Fig.A1(a).

- $\boldsymbol{PPI^{(1)}}$: On the other hand, a *1-step walk* on the PPI network can construct a new type of disease network—$\boldsymbol{PPI^{(1)}}$. In this network, an association between *Disease I* and *Disease II* is defined when the proteins are known to *interact* on the PPI network ($\boldsymbol{P_1} \sim \boldsymbol{P_2}$) although they are not identical ($\boldsymbol{P_1} \not\equiv \boldsymbol{P_2}$). Fig.A1(b) describes $\boldsymbol{PPI^{(1)}}$.

- $\boldsymbol{PPI^{(2)}}$: To define $\boldsymbol{PPI^{(2)}}$, let us introduce an extra protein $\boldsymbol{P_3}$ which is neighbored (or interacts) with the two proteins ($\boldsymbol{P_3} \sim \boldsymbol{P_1}, \boldsymbol{P_3} \sim \boldsymbol{P_2}$) and assume that there is no interaction between $\boldsymbol{P_1}$ and $\boldsymbol{P_2}$ ($\boldsymbol{P_1} \nsim \boldsymbol{P_2}$). See Fig.A1(c). To make a walk from $\boldsymbol{P_1}$ to $\boldsymbol{P_2}$, *two steps of walk* bypassing $\boldsymbol{P_3}$ are required so that $\boldsymbol{P_1} \rightarrow \boldsymbol{P_3} \rightarrow \boldsymbol{P_2}$ and vice versa. In $\boldsymbol{PPI^{(2)}}$, *Disease I* and *Disease II* are associated if they can be connected by a *2-step walk* on the PPI network.

- $\boldsymbol{PPI^{(3)}}$: In $\boldsymbol{PPI^{(3)}}$, a *3-step walk* connects *Disease I* and *Disease II.* As shown in Fig.A1(d), there is no direct interaction between $\boldsymbol{P_1}$ and $\boldsymbol{P_2}$ ($\boldsymbol{P_1} \nsim \boldsymbol{P_2}$) but there is between $\boldsymbol{P_3}$ and $\boldsymbol{P_4}$, proteins that interact with $\boldsymbol{P_1}$ and $\boldsymbol{P_2}$, respectively ($\boldsymbol{P_1} \sim \boldsymbol{P_3}$, $\boldsymbol{P_2} \sim \boldsymbol{P_4}$, $\boldsymbol{P_3} \sim \boldsymbol{P_4}$). Using these edges, a walk can be made $\boldsymbol{P_1} \rightarrow \boldsymbol{P_3} \rightarrow \boldsymbol{P_4} \rightarrow \boldsymbol{P_2}$, which leads to an association of *Disease I ~ Disease II.*

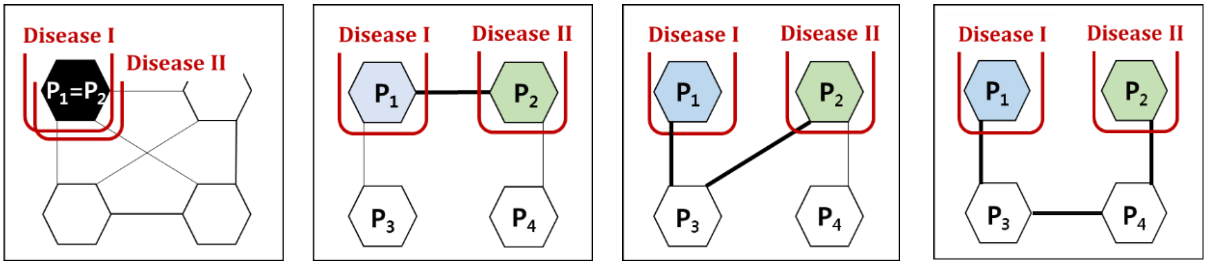

(a) $PPI^{(0)}$ (b) $PPI^{(1)}$ (c) $PPI^{(2)}$ (d) $PPI^{(3)}$

Figure I1. Association between diseases from a q-step walk on the PPI network: *Disease I* and *Disease II* are defined as *associated* (a) if P_1 and P_2 are identical, thus a *0-step walk* on the PPI network— $PPI^{(0)}$, (b) if P_1 and P_2 interact (*1-step walk*) — $PPI^{(1)}$, (c) if P_1 and P_2 are connected by a *2-step walk* ($P_1 \rightarrow P_3 \rightarrow P_2$)–$PPI^{(2)}$, and (d) if P_1 and P_2 are connected by a *3-step walk* ($P_1 \rightarrow P_3 \rightarrow P_4 \rightarrow P_2$)–$PPI^{(3)}$.
